# The telomere-to-telomere haplotype genome provides in-depth insights into the molecular mechanisms of the anthocyanin deficiency phenotype in *Prunus mume*

**DOI:** 10.1186/s43897-025-00186-8

**Published:** 2026-02-02

**Authors:** Pengyu Zhou, Xiao Huang, Wei Tan, Feng Gao, Yang Bai, Chengdong Ma, Ximeng Lin, Yufan Ma, Minglu Li, Zhaojun Ni, Ting Shi, Faisal Hayat, Jing Shao, Zhihong Gao

**Affiliations:** 1 College of Horticulture, Nanjing Agricultural University, Nanjing, Jiangsu China; 2 Department of Integrative Agriculture, College of Agriculture and Veterinary Medicine, United Arab Emirates University, Al Ain, United Arab Emirates; 3 Institute of Pomology, Jilin Academy of Agricultural Sciences (Northeast Agricultural Research Center of China), Changchun, China

**Keywords:** *Prunus mume*, T2T, Comparative genomics, Anthocyanin, *PmGSTF2*

## Abstract

*Prunus mume* originated from China and is highly valued for its remarkable combination of ornamental blossoms and high economic value. Previously published *P*. *mume* genomes contain unanchored genetic regions and excessive gaps. Here, we report the *P*. *mume* f. viridicalyx gap-free telomere-to-telomere (T2T) haplotype genome. The LE_hap1 and LE_hap2 genomes were 229.29 and 228.36 Mb in length, respectively, with an N50 length of the contig between 27.65 and 27.79 Mb and 24,318 and 24,316 protein-coding genes, respectively. The completeness, continuity, and accuracy of the *P*. *mume* f. viridicalyx genome was significantly improved over the previous *P*. *mume* genomes. The key mutated genes during *P*. *mume* f. viridicalyx cultivar domestication were identified by comparative genomic, population evolution, and selective sweep analyses to be significantly enriched in the anthocyanin metabolism process, glutathione metabolism process, and carotenoid biosynthesis. Further analysis revealed that early codon termination of the *PmGSTF2* gene, which is the key gene for the characteristic production of *P*. *mume* f. viridicalyx, reduced anthocyanin accumulation. We assembled a complete T2T gap-free haplotype *P*. *mume* genome, which provides a reference for gene mining and genome evolution of the anthocyanin deficiency phenotype in *P*. *mume*.

**Supplementary Information:**

The online version contains supplementary material available at 10.1186/s43897-025-00186-8.

## Core

We assembled a gap free telomere to telomere haplotype genome of *P. mume* f. viridylx, and found that the early codon termination mutation of *PmGSTF2* gene is a key regulatory gene leading to anthocyanin accumulation inhabitation of *P. mume* f. viridylx based on comparative genomics, population genetic evolution, and molecular biology evidences.

## Gene & accession numbers

The genome sequencing data have been deposited in the NCBI database under accession number is PRJNA1127312. The genome assemblies and annotation have been deposited in the Figshare database (DOI: 10.6084/m9.figshare.26105437). Information of gene in this study can be found in the NCBI database (https://www.ncbi.nlm.nih.gov/datasets/genome/?taxon=102107) under the accession numbers: *PmGSTF2* (LOC103327357).

## Introduction

Anthocyanins are naturally occurring pigments belonging to the flavonoid group and are widely distributed in higher plants, especially among fruit trees. Anthocyanidins are the primary pigments in many fruits, petals, and leaves and are responsible for providing plants with rich colours (Khoo et al. 2017). These colours help attract pollinating insects and seed-dispersing animals, thus promoting plant reproduction and seed dispersal. It also enables them to withstand adverse environments. When plants are exposed to adversities, such as bright light, low temperatures, and drought, they can survive by stressfully synthesising anthocyanidin glycosides, which protect the plant from bright light, ultraviolet rays, and low temperatures (Li and Ahammed 2023).

Anthocyanin biosynthesis can be roughly divided into three stages: phenylpropane metabolism pathway, flavonoid metabolism pathway, and anthocyanin synthesis modification pathway. Currently, many genes, including those encoding *MYB* (Gu et al. 2019; Rouholamin et al. 2015; Xu et al. 2017; Xu et al. 2020; Yamagishi et al. 2010), *bHLH* (Hichri et al. 2010; Liu et al. 2013), and *WD40* (Ben-Simhon et al. 2011; Li 2014), which regulate the expression of anthocyanin biosynthesis-related genes by binding to structural gene promoters, have been identified to participate in the anthocyanin pathway. The transport and accumulation of anthocyanins greatly affect the color phenotype of plants. After synthesis in the endoplasmic reticulum, anthocyanins are then transported via vesicular transport with transporter proteins, such as glutathione S⁃transferase (*GST*), multidrug toxicity exclusion transporter (*MATE*), and ATP⁃binding cassette (*ABC*), to the vesicles for storage. Glutathione transferase (*GST*) is an anthocyanin transporter protein that transports anthocyanins into the vesicles in fruit and flowers. Glutathione S-transferase (*GST*) catalyses the binding of anthocyanins to glutathione through covalent bonds to form glutathione S-conjugates. The vacuolar membrane has the activity of a glutathione S-binding pump (GSH-S-X), recognising glutathione and transporting it to the vacuoles to form anthocyanin vesicular inclusions (AVIs), thereby completing anthocyanin fixation, accumulation, and storage. Due to its special affinity activity, glutathione S-transferase is considered to play a key role in pigment transportation and accumulation and is the last enzyme in anthocyanin biosynthesis. In apple, the transcription factor *MdMYB1* promotes the transfer of anthocyanins to vesicles by binding to the *MdGSTF6* promoter (S Jiang et al. 2019). Knockdown of the *GST* gene hinders anthocyanin transport in strawberry plants, causing the stems to turn green and the fruit to turn white (Gao et al. 2020). In peach, functional loss caused by mutations in the *GST* gene leads to a decrease in anthocyanins in the pericarp (Lu et al. 2021).

*Prunus mume* is a deciduous tree of the Rosaceae family and is native to China, with current cultivation mainly concentrated in subtropical areas (Hayat et al. 2021; Huang et al. 2022). *Prunus mume* germplasm is extremely rich in resources and produces a variety of anthocyanin-associated phenotypes in stems, buds, leaves, pericarp, endocarp, and flowers, making it highly ornamental. *Prunus mume* usually has dark purple sepals, but some species have green sepal, with *P*. *mume* f. viridicalyx being the best known (Xie 1978). It belongs to the class of straight-branched *P*. *mume* varieties with single or compound petal flowers that are light green at first bloom and white at full bloom, with green sepals, branches, and leaves, which are quite different from other *P*. *mume* varieties (Zhang 2007). It not only adds more ornamental types to *P. mume* varieties, but also has high recognition among many *P. mume* varieties, with green sepals, white flowers, and green twigs. This uniqueness increases the ornamental charm of green sepal *P. mume* (Wang et al. 2024). Moreover, the chlorogenic acid content is high in the green calyx of *P*. *mume*; therefore, it has an extremely high medicinal value (Wang et al. 2024).

Assembling high quality genomes is a common approach for in-depth understanding of a species. With the advent of accurate long-read sequencing technologies, the concept of telomere-to-telomere (T2T) has been brought to the forefront of plant genome research (Garg et al. 2024). A complete T2T genome contains all the genomic information of a species and can be regarded as the ultimate goal of genome assembly. It can avoid mapping errors and improve the accuracy of variant detection; identify genes and genetic information overlooked in previous studies, and provide more accurate haplotype genomic information (Lan et al. 2023). By accurately resolving repetitive sequences, the T2T genome reveals the structures of centromeres and telomeres, and helps to annotate more protein-coding genes, opening up new avenues for the development of comparative genomics and evolutionary biology (Alkan et al. 2022). So far, the genomes of multiple species have been reported as gap-free or T2T genomes, such as flowering cherry (Jiang et al. 2025), carnation (Lan et al. 2023), kiwifruit (Han et al. 2023), and rosa (Zhou et al. 2024). However, the T2T genome of *Prunus mume* has not been reported yet. In this study, we constructed a haplotype T2T genome for *P*. *mume* f. viridicalyx, as the object of study, and analysed its evolutionary and genomic features. Combined with the comprehensive analysis of resequencing data, new evidence for the absence of anthocyanins in *P*. *mume* f. viridicalyx was found. The present work provides a solid theoretical foundation and scientific basis for the study of anthocyanin synthesis in *P*. *mume*.

## Results

### A telomere-to-telomere haplotype genome for *Prunus mume* f. viridicalyx

**Fig. 1:**
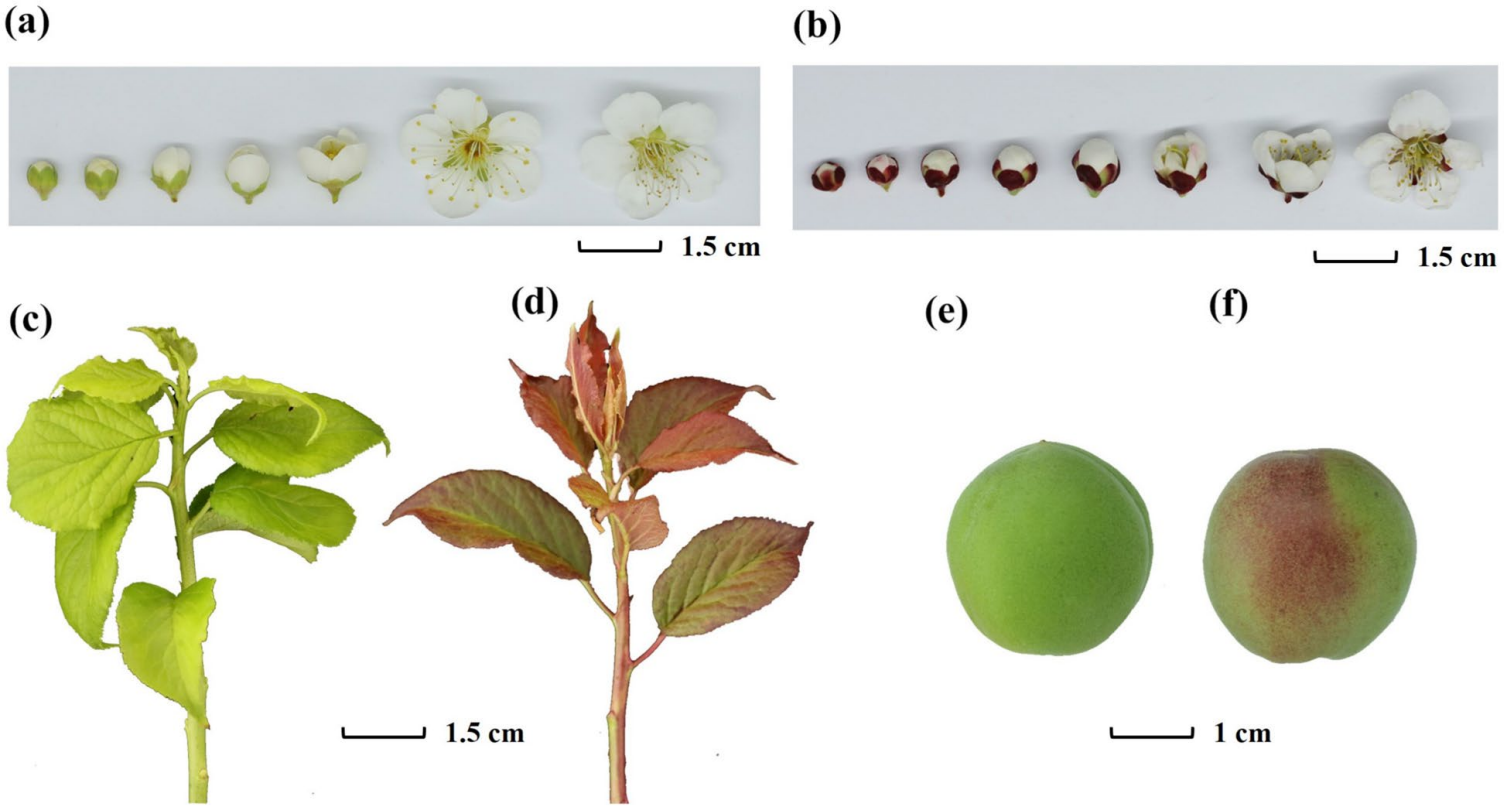
Phenotypic pictures of *Prunus mume* f. viridicalyx cv Lv E (LE) and *Prunus mume* cv Ruantiao Hongmei (RTHM). **a** Flower phenotypes at different stages of LE and (**b**) RTHM. **c** Leaf and branch phenotypes of LE and (**d**) RTHM. **e** Fruit phenotypes at the mature stage of LE and (**f**) RTHM

*Prunus mume* f. viridicalyx belongs to the class of straight *P*. *mume*. The flowers are single or compound, light green at the beginning of blooming, white when in full bloom, and the sepals are green. The flowers are large and beautiful, and the leaves and branches of the plant are green, giving it a high ornamental and application value. Compared to other *P*. *mume* varieties, the sepals and young leaves almost showed a purple or purple-red colour (Fig. 1).

**Fig. 2:**
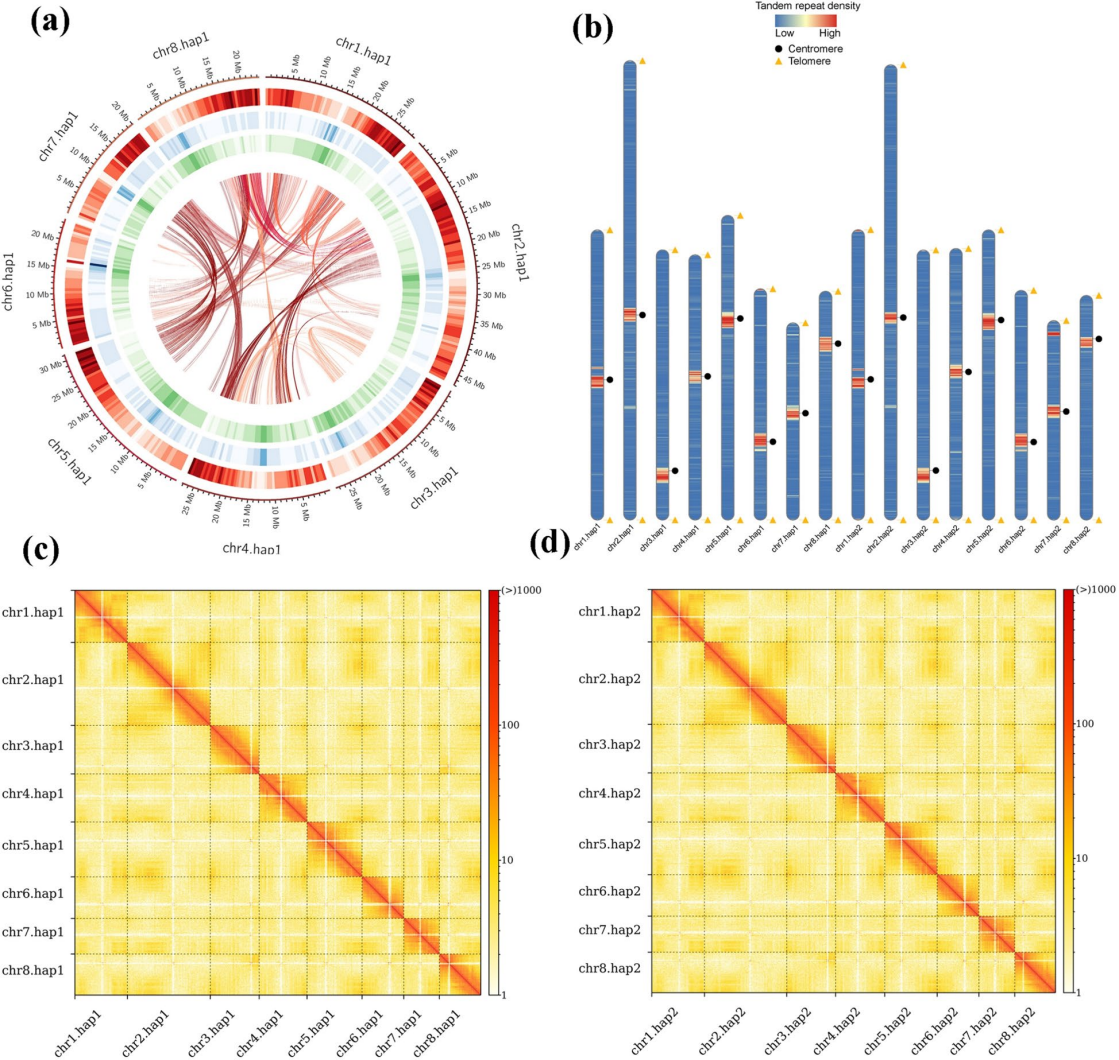
*Prunus mume* f. viridicalyx genome features, and synteny information. **a** Overview of the LE_hap1 draft genome assembly. From the outside to the inside: Chromosome; Gene; GC content; Repeat sequence. **b** Telomere and centromere detection map. Orange triangles and purple circles represent telomeres and centromeres, respectively, within the assembled genome; blue indicates a low gene density; and red indicates a high gene density. **c** Hi-C interaction heatmap of LE genome haplotype 1; (d) Hi-C interaction heatmap of LE genome haplotype 2

We incorporated multiple sequencing techniques to assemble the genome from telomere to telomere. Based on Illumina short reads, the estimated genome size was 224.70 Mb. Based on the PacBio Sequel II platform, we obtained clean HiFi reads of 26.08 GB, with an N50 length of 14.29 kb and a sequencing depth of 116.05 ×. We used Oxford nanopore technology (ONT) to generate an ultra-long read of 27.00 Gb, with an N50 and sequencing coverage of approximately 100.00 kb and 120.14 × (Table S1). During the assembly process, we used high-quality HiFi reads and ONT ultra-long reads for mixed assembly (Table S2) with hifiasm software (0.19.6, https://github.com/chhylp123/hifiasm). Subsequently, the contigs were further ordered, oriented, and grouped using Hi-C reads, enabling their anchoring to the chromosome level (Tables S3 and S4, Fig. 2c and d). Two haplotype T2T genomes were generated, with sizes of 229.29 and 228.36 Mb (Table 1). The contig N50 values were 27.65 and 27.79 Mb, and the longest contigs were 47.06 and 46.03 Mb (Table 1). The basic structural features of the genome are shown in Fig. 2a.

**Table 1:** Major indicators of *Prunus mume* f. viridicalyx and *P*.* mume*

	*Prunus mume f. viridicalyx* haplotype 1	*Prunus mume f. viridicalyx* haplotype 2	*Prunus mume* var.* tortuosa*	*Prunus mume* (wild type)
Assembly size (Mb)	229.99	228.36	262	280
Total number of contigs	8	8	225	45,811
Total length of contigs (Mb)	229.99	228.36	237.7	219.9
Contig N50	27.65 Mb	27.79 Mb	2.75 Mb	31.8 kb
Contig N90	23.42 Mb	22.99 Mb	546.9 kb	5.77 kb
Longest contig (Mb)	47.03	46.60	12.6 Mb	—
GC content (%)	37.74	37.74	37.46	—
Complete BUSCOs (%)	98.90	98.90	96.40	—
Number of annotated genes	24,318	24,316	26,015	25,905
Average gene length (bp)	3245	3084	3897	2514
Average length of coding sequences (bp)	1360	1344	1258	1146
Repeat content (%)	43.05	42.76	52.12	45
Gap number	0	0	104,500	16,851,380

We used the telomere sequence of CCCTAAA at the 5-terminus or TTTAGGG at the 3-terminus as a query to scan the whole genome of ‘LE_hap1’ and ‘LE_hap2’. A total of 32 telomeres on 16 chromosomes were identified in each haplotype genome, ranging in size from 1070 to 2245 bp (Fig. 2b, Table S5). We also identified 16 possible centromeres in each haplotype genome, with sizes ranging from 1,325,595 to 4,387,690 bp (Fig. 2b, Table S6).

### Quality assessment and annotation of the telomere-to-telomere genome

We used Benchmarking Universal Single-Copy Orthologs (BUSCO) to evaluate the completeness of the assembly and annotation. There were 98.9% of core eukaryotic genes in the *P*. *mume* f. viridicalyx haplotype 1 (LE_hap1) and *P*. *mume* f. viridicalyx haplotype 2 (LE_hap2) genomes, with 23 duplicated orthologous genes and 1574 single-copy orthologous genes in LE_hap1 and 26 duplicated orthologous genes and 1571 single-copy orthologous genes in LE_hap2 (Table S7). The genomic continuity, as assessed by the long terminal repeat (LTR) assembly index (LAI), showed values of 23.01 (Table S8) and 22.68 (Table S9) for the LE_hap1 and LE_hap2 genomes, respectively, reaching the “gold standard” level (LAI > 20). The consensus quality value (QV) of the genome also showed that all chromosomes had QV values over 60 in the LE_hap1 genome (Table S10). In the LE_hap2 genome, the QV value of only one chromosome was 53.43; all other chromosomes had QV values over 60 (Table S11), indicating that the whole genome was assembled with a very high degree of accuracy. In conclusion, these results indicate that the haplotype T2T genomes of LE_hap1 and LE_hap2 all reached a high level of accuracy, completeness, and continuity.

Based on de novo prediction, homology prediction, and transcript prediction, we predicted 24,318 protein-coding genes in the LE_hap1 genome and analysed their lengths. The average gene, CDS, exon, and intron lengths were 3254, 1360, 321, and 361 bp, respectively. We predicted 24,316 protein coding genes in the LE_hap2 genome and analysed their lengths. The average gene, CDS, exon, and intron lengths were 3084, 1344, 299, and 354 bp, respectively, which is consistent with the predicted results of other *Prunus* plants (Table S12, Figure S1). In addition, we identified 3281 and 3144 non-coding RNAs, mainly composed of 351 and 301 ribosomal RNAs, 479 and 508 transfer RNAs, and 1856 and 1789 snRNAs, snoRNAs, and microRNAs in the LE_hap1 and LE_hap2 genomes, respectively (Table S13). We used RepeatModeler to perform repeat sequence de novo prediction and then merge the predicted results with the RepBase database. In the assembled genome sequence, 99.00 and 97.64 Mb were identified as a repetitive sequence, accounting for approximately 43.05 and 42.76% of the LE_hap1 and LE_hap2 genome sizes, respectively (Table S14).

### Comparative evolutionary analyses of *Prunus mume* f. viridicalyx and other Prunus species

**Fig. 3:**
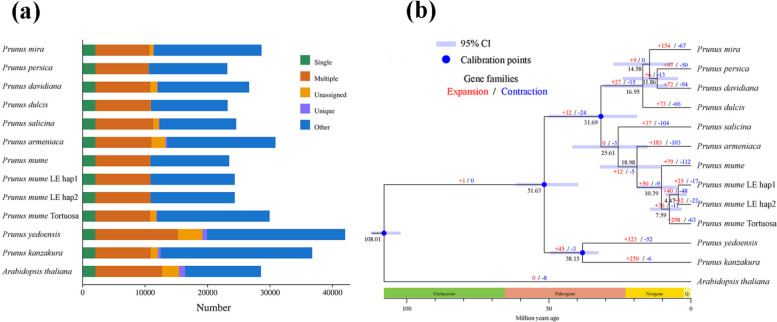
Evolution of the *Prunus mume* genome and gene families. **a** The distribution of single-copy, multiple-copy, unique, unassigned, and other orthologs in the 12 plant species. **b** Phylogenetic tree of 12 plant species. The black numbers denote the divergence time of each node (MYA, million years ago), blue nodes indicate calibration points, and light blue indicates the 95% confidence intervals. The numbers on each branch of the tree represent the total number of expanded (red) and contracted (blue) gene families

We used LE_hap1, LE_hap2 and 11 other sequenced species to identify the putative orthologous gene clusters based on paired sequence similarity. A total of 24,318 and 24,316 genes were identified, including 2092 and 2092 single-copy genes, 8767 and 8780 multicopy genes, and 13,396 and 13,407 other orthologs genes in the LE_hap1 and LE_hap2 genomes (Fig. 3a). However, there were 14,038 gene families in *P*. *mume* f. viridicalyx, wild *P*. *mume*, *P*. *mume* var. tortuosa, *P*. *armeniaca*, and *P*. *salicina* and 156 unique gene families in the *P*. *mume* LE_hap1 and LE_hap2 genomes (Figure S2). The specific genes subsequently underwent Gene Ontology (GO) analysis for functional annotation analysis and Kyto Encyclopaedia of Genes and Genomes (KEGG) database analysis to identify the involved enriched pathways. The GO annotation results showed that the unique genes of *P*. *mume* f. viridicalyx were mainly involved in response to stimulus, biological regulation, and multi-organism processes in the biological processes and antioxidant activity, nutrient reservoir activity, and transporter activity in the molecular functions (Figure S3). In addition, genes related to the carotenoid biosynthetic process were significantly enriched. For the KEGG pathways, the top enriched KEGG pathways were amino acid synthesis and metabolism, glutathione metabolism, and glycolysis/gluconeogenesis (Figure S4).

Analysis of gene family expansion and contraction showed that compared to the gene families of its close relative *P*. *mume*, *P*. *mume* f. viridicalyx was significantly enriched and reduced by 40 and 48 gene families, respectively (Fig. 3b). To study the phylogenetic relationships of *P*. *mume* f. viridicalyx, we selected the single-copy genes from 11 *Prunus* genomes and an outgroup genome, *Arabidopsis thaliana*, to construct a phylogenetic tree. Three *P*. *mume* clustered together, with *P*. *mume* f. viridicalyx and *P. mume* var. tortuosa more closely related compared to wild *P*. *mume*. The ancestor of *P*. *mume* f. viridicalyx and *P. mume* var. tortuosa diverged from wild *P*. *mume* approximately 7.59 million years ago (Mya); later, about 4.47 million years ago, *P*. *mume* f. viridicalyx and *P. mume* var. tortuosa diverged (Fig. 3b).

### Comparative and evolutionary analysis of TEs in different *Prunus mume* species

In the LE_hap1 and LE_hap2 genomes, 43.05 and 42.76% of genome sequences were annotated as repetitive elements, with transposon elements (TE) accounting for 80.26 and 79.96%, respectively (Table S15). Long-terminal repetitive reverse transcription transposons (LTRs) were the main category of TE, accounting for 22.70 and 22.29% of the genome. In LTR, LTR/Gypsy elements were the most abundant, accounting for 15.44 and 15.04% of the genome, followed by LTR/Copia elements, accounting for 5.77 and 5.77% of the genome. We further carried out a comparative analysis of transposon information in the three *P*. *mume* genomes, which showed that the number (Figure S5), proportion (Figure S6), and total length (Figure S7) of the different transposons were significantly lower in wild *P*. *mume* than in *P. mume* f. viridicalyx and *P. mume* var. tortuosa. The distribution of LTRs along the chromosome (Figure S8) showed the same results.

**Fig. 4:**
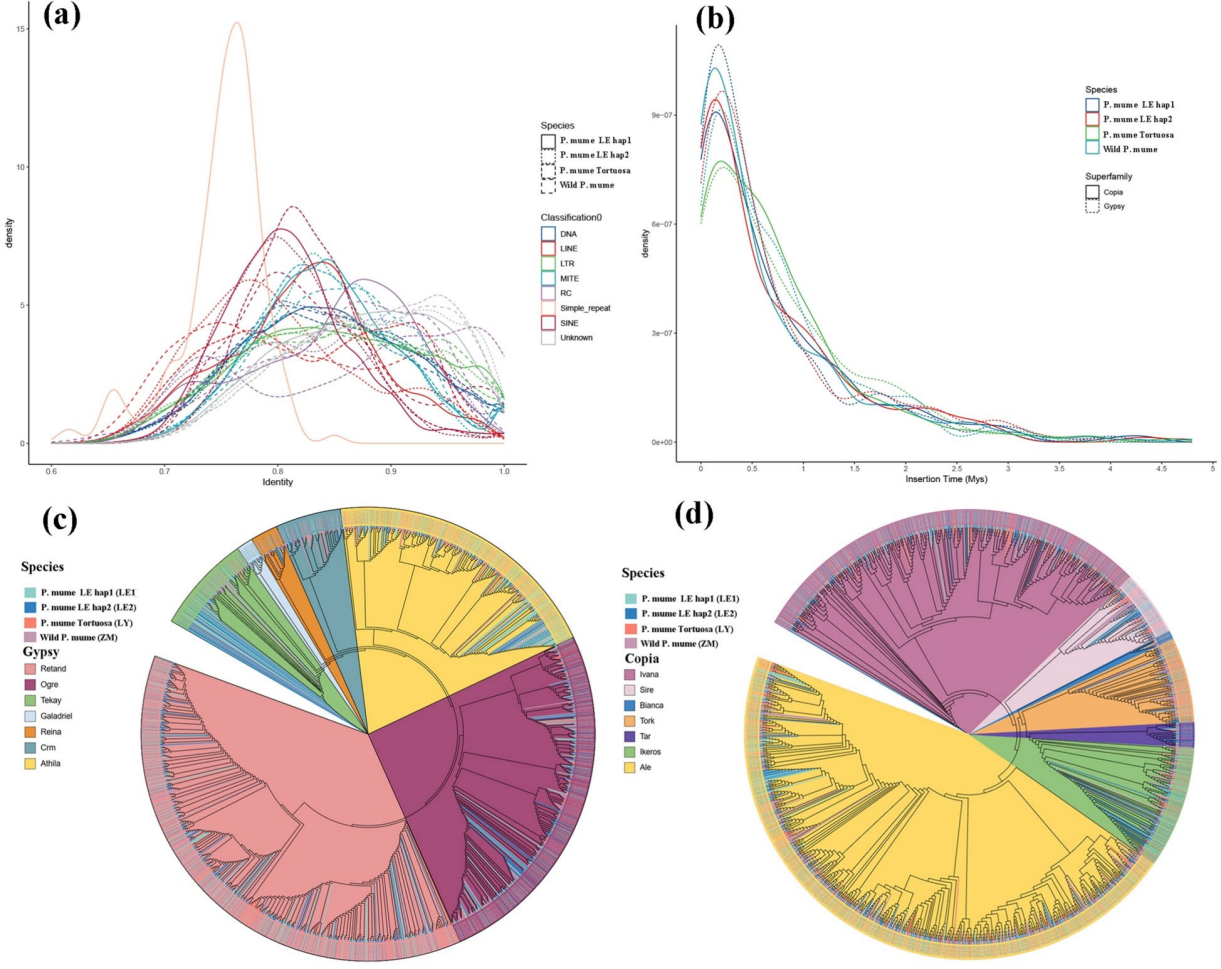
TE analysis in the wild *Prunus mume*, *P. mume* f. viridicalyx and *P. mume* var. tortuosa genomes. **a** Distribution of sequence identity values between genomic copies and consensus repeats in the genomes. The relative frequencies per percentage of identity of the DNA, MITE, Pararetrovirus, Polinton, LTR, LINE, and unknown TEs are represented in different colours. **b** Estimated insertion times of LTR retrotransposons in the genomes. **c** Phylogenetic tree of Ty1/copia LTR retrotransposons in the genomes. **d** Phylogenetic tree of Ty3/gypsy LTR retrotransposons in the genomes

To determine the genetic evolution history of TEs in the *P. mume* f. viridicalyx genome, we analysed the identity values between genome copies and their shared sequences (Fig. 4a). The peaks of the distributions differed among repeat classes. Among these, the first peak of the LINE element was around 75%, and its appearance was also the earliest peak, indicating that it was relatively old. In addition, we observed its second peak, whose identity was around 95%, indicating a recent outbreak. We further evaluated the insertion time of the LTR and showed that the wild* P*. *mume*, *P*. *mume* LE_hap1, *P*. *mume* LE_hap2, and *P*. *mume* var. tortuosa genomes had a major burst of Copia and Gypsy elements 0.25 million years ago. In contrast, the wild *P*. *mume* genome had a minor burst of Copia elements 0.25 million years ago and a major burst of Gypsy elements 2 million years ago (Fig. 4b). To study the evolution of TEs in *P*. *mume* f. viridicalyx, we constructed phylogenetic trees of the Ty1/Copia and Ty3/Gypsy superfamily. The phylogenetic tree of the Ty3/Gypsy superfamily showed that most LTR-RTs of wild *P*. *mume* clustered into the RETAND and CRM branches. In contrast, the LTR_RTs of *P*. *mume* ‘LE’ and Tortuosa had a uniform distribution in all seven branches (Fig. 4c), showing higher diversity and abundance, suggesting that the *P*. *mume* f. viridicalyx and *P*. *mume* var. tortuosa genomes have more significant expansion and differentiation. The Copia superfamily showed a different evolutionary pattern, with element distributions of all three species in all five major branches (Fig. 4d), suggesting that the Copia superfamily has a conserved evolutionary pattern.

### Whole-genome duplication event and positively selected genes in the genome

To further study the whole genome duplication events of *P. mume* f. viridicalyx, we calculated the fourfold degenerate synonymous site (4DTV) and synonymous mutation (Ks) rates. The 4DTV and Ks values peaked at 0.03 for paralog pairs in *P*. *mume* f. viridicalyx, highlighting the recent whole-genome duplication in this species, similar to other *Prunus* species (Figure S9). We also explored the genome syntenic blocks between *P*. *mume* f. viridicalyx and the other representative *P*. *mume* species. Our genome assembly of *P*. *mume* f. viridicalyx exhibited a high level of genome synteny with all other *P*. *mume* genomes, indicating that the genome we assembled has high quality (Figure S10).

The Ka/Ks density curves showed little difference in the values for *P*. *mume* f. viridicalyx and other *Prunus* plants. To further explore the genes subjected to positive selection in *P*. *mume* f. viridicalyx, we focused on analysing the Ka/Ks values of *P*. *mume* f. viridicalyx with two other *P*. *mume* varieties (Figure S11a). There were 927 pairs of homologous genes with a Ka/Ks value over 1 between LE_hap1 and wild *P*. *mume* and 1109 pairs between LE_hap1 and *P*. *mume* var. tortuosa. A total of 163 pairs of genes were shared among these positively selected genes (Figure S11b). We further conducted enrichment analysis on these positive selection genes, and many genes involved in stress response to different types of stimuli were significantly enriched, mainly including water deprivation, salt, viruses, DNA damage, and oxidative stress (Figure S11c). In addition, in the KEGG enrichment pathway, we found significant enrichment of genes involved in carotenoid biosynthesis and amino acid metabolism (Figure S11c). There were 913 pairs of homologous genes with a Ka/Ks value over 1 between LE_hap2 and wild *P*. *mume* and 1050 pairs between LE_hap2 and *P*. *mume* var. tortuosa. A total of 143 pairs of genes were shared among these positively selected genes (Figure S11b). These genes are involved in scopoletin glucosyltransferase activity, pentose and glucuronate interconversions, sulphate adenylyl transferase (ADP) activity, and terpenoid backbone biosynthesis (Figure S11d).

### Global comparison genomic analysis between the *P. mume *f. viridicalyx and wild-type genome

**Fig. 5:**
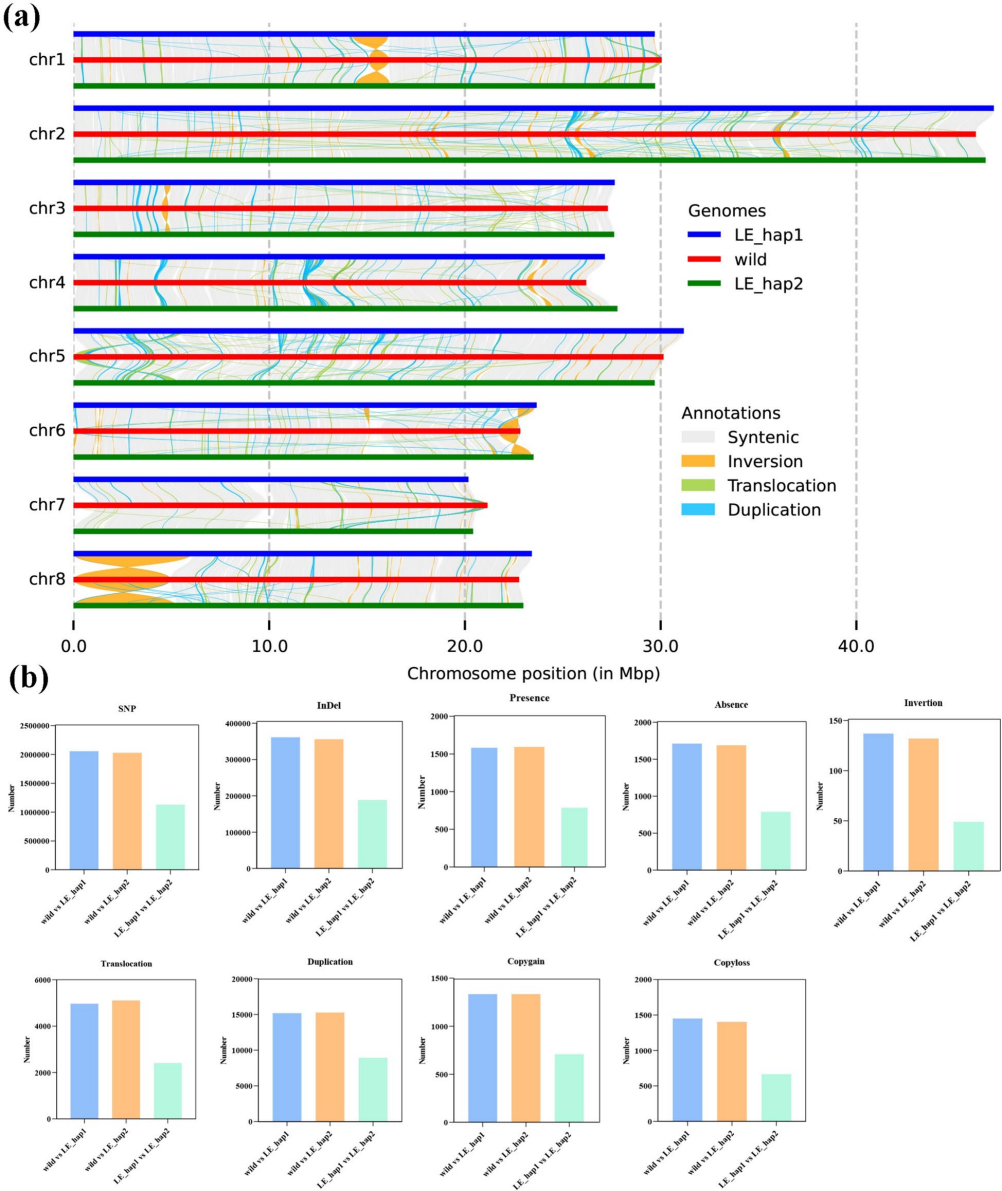
Collinearity and variation analysis of *P. mume* f. viridicalyx and wild *Prunus mume* genomes. **a** Collinearity analysis between ‘LE’ and wild *P*. *mume* genomes. The wild genome was set as a reference. **b** Distribution of the number of SNP, InDel, presence, absence, inversion, translocation, duplication, copy gain, and copy loss variations in different genomes

To explore the molecular mechanism of domestication of *P*. *mume* f. viridicalyx, we conducted comparative genomic analysis (Fig. 5a) using wild *P*. *mume* as a reference genome (Wang et al. 2021). In the LE_hap1 genome, 342 specific genes were identified (Table S15), mainly enriched in the starch and sucrose metabolism, oxidative phosphorylation, biosynthesis of unsaturated fatty acids, and cyanoamino acid metabolism pathways (Figure S12). In the LE_hap2 genome, 341 specific genes were identified (Table S16), mainly enriched in the starch and sucrose metabolism, oxidative phosphorylation, biosynthesis of unsaturated fatty acids, and cyanoamino acid metabolism pathways (Figure S13). In the wild-type genome, 80 specific genes were identified (Table S17 and Figure S14); these genes were mainly enriched in plant hormone signal transduction, polysaccharide binding, diacylglycerol cholinephosphotransferase activity, metal ion binding, and monooxygenase activity (Figure S15).

We also detected genomic variations, with 2,055,970, 2,027,412, and 1,130,137 SNPs and 361,196, 356,031, and 188,624 InDels mainly distributed in the inter-gene (43.93–46.66%), upstream gene (15.70–15.99%), and intron regions (12.57–14.55%), with the exon region only accounting for 5.05–5.55% (Table S18). In the gene-coding region, SNP mutation types included non-synonymous (50.23–50.89%), synonymous (43.90–44.10%), and relatively large stop codon mutations (0.98–1.34%). The main mutation types in InDels were frameshift (2.15–2.61%) and non-frameshift mutations (1.80–1.90%) (Table S19). The structural variation results identified 14,262 syntenic regions, 137 inversions, 4968 translocations, 15,178 duplications, and 12,177 unaligned regions based on structural annotations between wild and LE_hap1 genomes (Table S20). A total of 1608 insertions, 1711 deletions, 1334 copy gains, 1450 copy losses, 3177 highly diverged, and 462 tandem repeats were identified based on sequence annotations between wild and LE_hap1 genomes (Fig. 5B and Table S20). The structural variation results identified 14,217 syntenic regions, 132 inversions, 5106 translocations, 15,263 duplications, and 12,443 unaligned regions based on structural annotations between wild and LE_hap2 genomes (Table S21). A total of 1594 insertions, 1689 deletions, 1323 copy gains, 1402 copy losses, 3279 highly diverged, and 496 tandem repeats were identified based on sequence annotations between wild and LE_hap2 genomes (Fig. 5B and Table S21). The structural variation results identified 7716 syntenic regions, 49 inversions, 2406 translocations, 8942 duplications, and 6289 unaligned regions based on structural annotations between LE_hap1 and LE_hap2 genomes (Table S22). A total of 812 insertions, 789 deletions, 709 copy gains, 667 copy losses, 1490 highly diverged, and 407 tandem repeats were identified based on sequence annotations between the LE_hap1 and LE_hap2 genomes (Fig. 5b and Table S22).

Furthermore, 1601–3319 presence/absence variations (PAVs) were identified among 3 genomes, and their distribution areas were primarily in the upstream and downstream regions of genes (36.29–37.71%), intergenic regions (40.33–43.72%), and intron regions (13.24–15.55%), with exon regions only accounting for 3.68–4.37% (Table S23). The mutation types included frameshift (60.00–62.30%), non-frameshift (13.93–17.14%), and termination codon acquisition mutations (22.86–23.77%) (Table S24).

### Population structure and genetic differentiation analysis

**Fig. 6:**
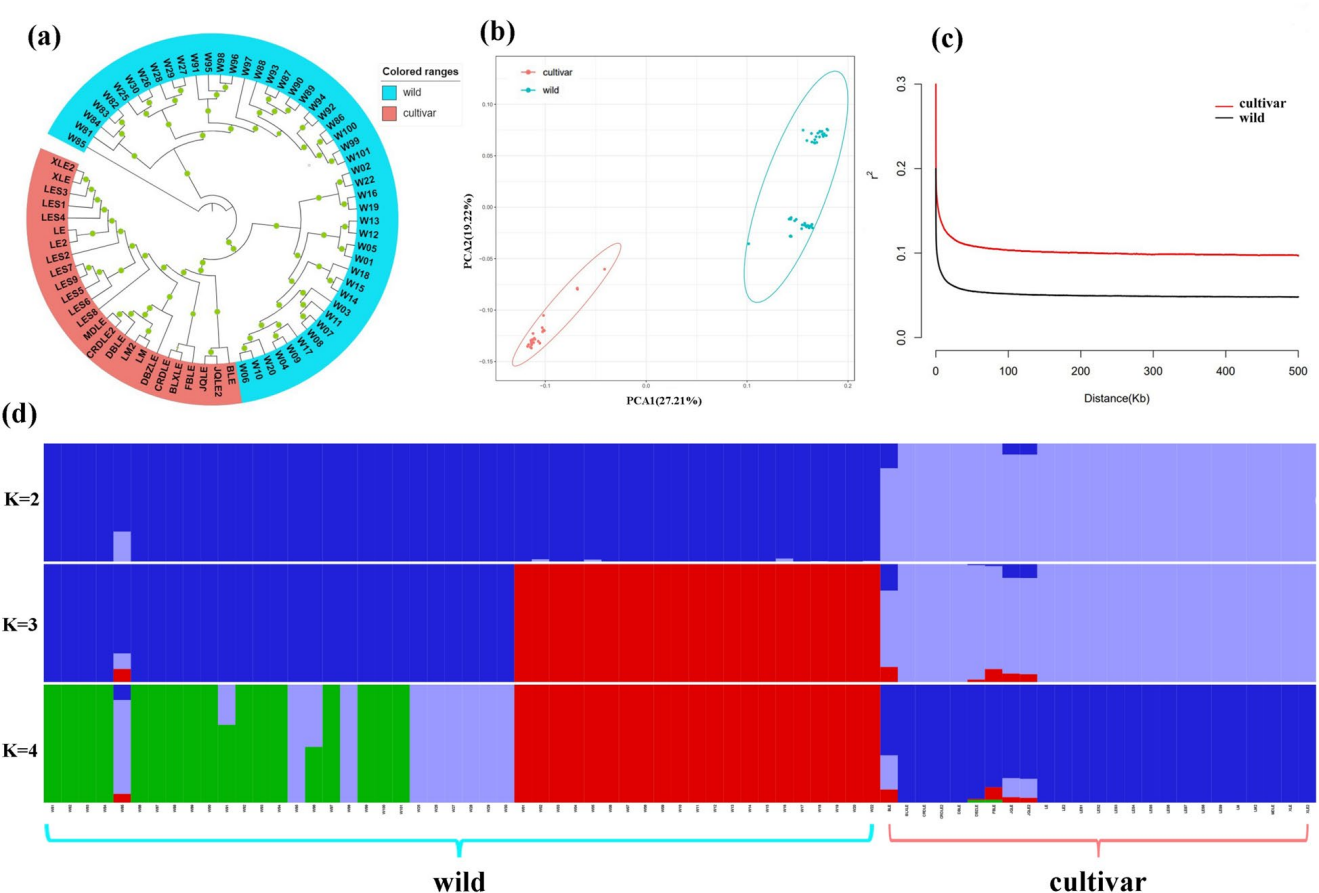
Population structure of *Prunus mume* germplasm resources. **a** Phylogenetic tree established by SNPs based on maximum likelihood; (**b**) Principal component analysis (PCA) of the *P*. *mume* accessions; (**c**) Linkage disequilibrium decay comparison among two *P*. *mume* groups; (**d**) Population structure analysis of the *P*. *mume* accessions

To further explore the anthocyanin synthesis genes involved in the green sepal-type *P*. *mume*, we selected wild samples and green sepal-type samples for genome-wide resequencing. We constructed a phylogenetic tree of 74 *P*. *mume* germplasm resources (Fig. 6a), which was generally divided into two major branches, one containing wild samples and the other containing green sepal-type samples. The 73 materials collected were analysed using principal component analysis (PCA) (Fig. 6b) and PCA scores to evaluate genetic variation. The principal components (PCA1 and PCA2) accounted for 27.21 and 19.22% of all variation, respectively. The wild samples formed closely related clusters, while the green sepal-type samples formed a single cluster. The population structure results also showed that when K = 2, the wild and green sepal-type samples were clearly separated (Fig. 6d). The strength analysis of linkage disequilibrium for the two subgroups was further examined (Fig. 6c). The linkage disequilibrium index r^2^ of both the cultivated group (green sepal-type sample) and the wild group was weakened at 10 bp. After approximately 8.6 kb of SNPs, the r^2^ of the cultivation group decayed to half the maximum r^2^ value. However, the r^2^ of the wild group decreased to half the maximum r^2^ value after approximately 1.1 kb of SNPs. The populations with green sepal-type samples had high linkage strength, slow linkage disequilibrium decay, and a long decay distance.

We conducted selective sweep analysis to explore potential candidate genes involved in the domestication of green sepal-type *P*. *mume*. Based on the F_ST_ value, selective sweep analysis identified 108 selected regions. This region was 19.62 Mb in size and contained 2390 genes (Figure S16a). The Pi value of selective sweep analysis identified 68 selected regions. The size of this region was 14.41 Mb and contained 1463 genes (Figure S16b). We combined the screening of genes with high-impact variation regions from comparative genomes. A total of 14 candidate genes were identified as potential candidate genes using 3 methods (Figure S16c). Enrichment analysis showed that many genes involved in abiotic stress, including temperature, radiation, salt, osmotic stress, toxins, DNA damage, and biological stimulation, were significantly enriched (Figure S16d). Secondary metabolite synthesis, glutathione metabolism, and glycolytic genes were significantly enriched in the KEGG metabolic pathway (Figure S16e).

### SNP variation analysis of candidate genes

**Fig. 7:**
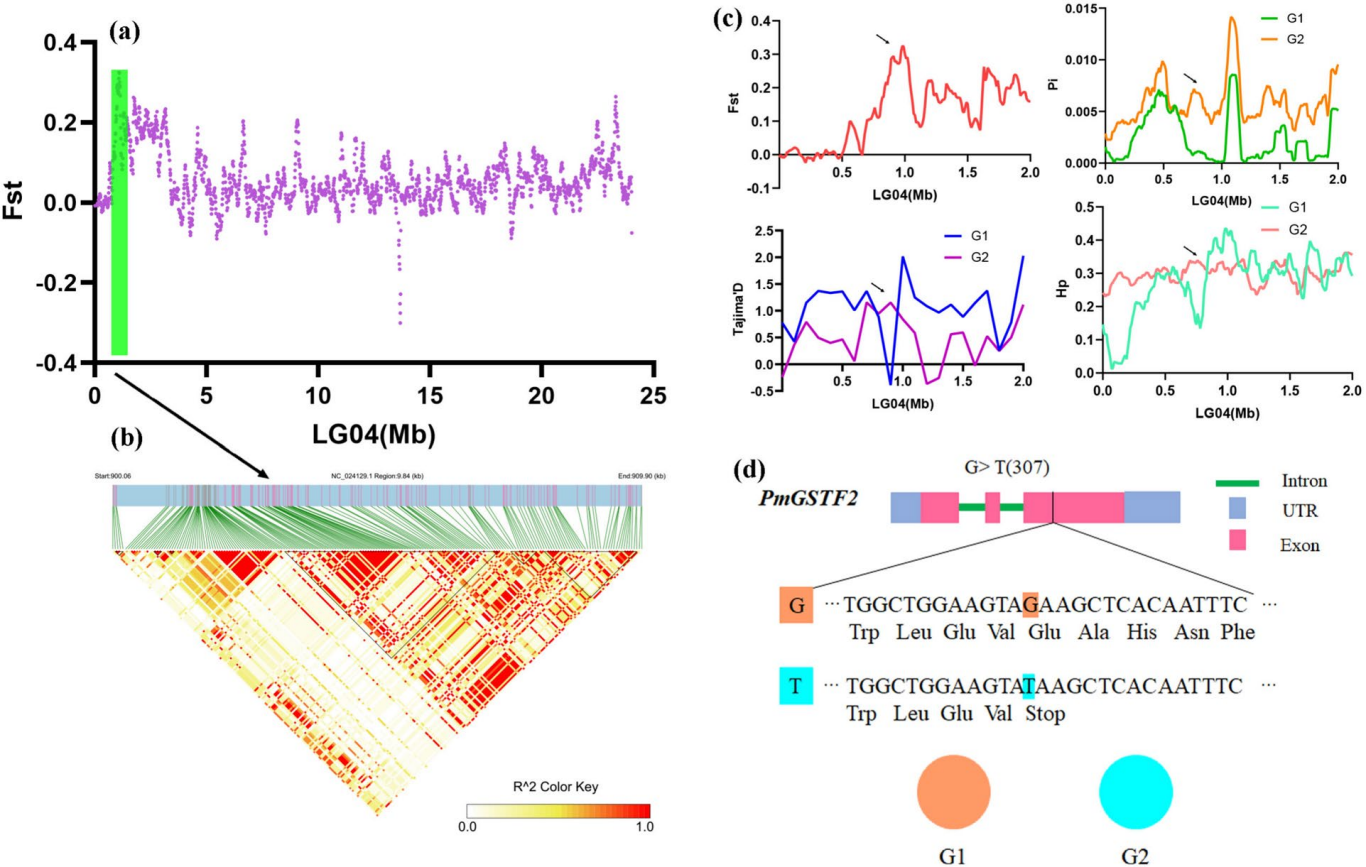
Analysis of genomic regions and genes under selection on chromosome 4. **a** Manhattan plots showing the results of selective sweep analysis of chromosome 4. **b** Peak regions on chromosome 4 are shown with the LD blocks. **c** Distribution of F_ST_, Pi, Tajima’s D, and Hp in the region around *PmGSTF2* on chromosome 4 (1.08–1.10 Mb). **d** Structure of the *PmGSTF2* gene and allelic frequencies of the associated SNP in *PmGSTF2* between two populations. **** indicates extremely significant differences between the two populations

On chromosome 4, one highly selected and highly linked region, LG04-LD1 (1.08–1.10 Mb), was identified (Fig. 7). A glutathione S-transferase *PmGSTF2* gene located on the fourth chromosome of *P*. *mume* was found. We further analysed the variation of the *PmGSTF2* gene and found that there was a codon early termination mutation in the *PmGSTF2* gene that mainly occurred in the *P. mume* f. viridicalyx samples population; no such early termination mutation was observed in the wild *P. mume* samples population (Fig. 7d).

### Overexpression and knockout of *PmGSTF2* gene regulated anthocyanin accumulation

**Fig. 8:**
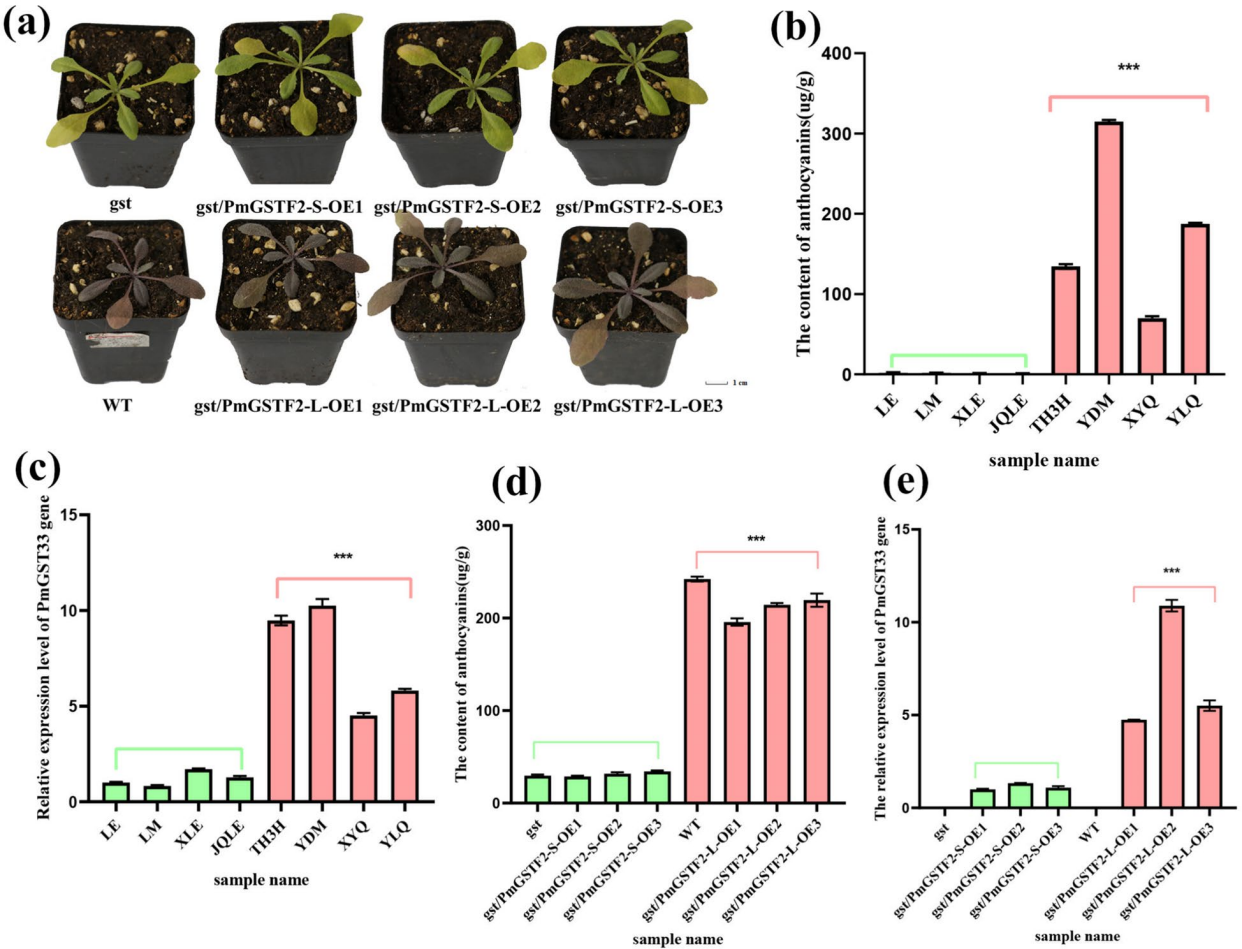
Functional analysis of the *PmGSTF2* gene. **a** Phenotypes of wild-type, *gst* mutant, gst/*PmGSTF2*-S, and gst/*PmGSTF2*-L transgenic strains of *Arabidopsis thaliana* under light treatment. **b** The anthocyanin content in green (‘LE’, ‘LM’, ‘XLE’, and ‘JQLE’) and non-green sepal-type samples (‘TH3H’, ‘YDM’, ‘XYQ’, and ‘YLQ’) of *Prunus mume*. LE: Lv E; LM: Lv mei; XLE: Xiao Lve; JQLE: Jinqian Lve; TH3H: Taihu No.3; YDM: Yadan Mei; XYQ: Xiye Qing; YLQ: Yeli Qing. **c** Expression level of the *PmGSTF2* gene in green (‘LE’, ‘LM’, ‘XLE’, and ‘JQLE’) and non-green sepal-type samples (‘TH3H’, ‘YDM’, ‘XYQ’, and ‘YLQ’) of *P*. *mume*. **d** Anthocyanin content in wild-type, *gst* mutant, gst/*PmGSTF2*-S, and gst/*PmGSTF2*-L transgenic strain samples of *Arabidopsis thaliana*. **e** Expression level of the *PmGSTF2* gene in wild-type, *gst* mutant, gst/*PmGSTF2*-S, and gst/*PmGSTF2*-L transgenic strain samples of *Arabidopsis thaliana*

Four green and non-green sepal-type samples were randomly selected, and the *PmGSTF2* gene was cloned and sequenced for verification (Figure S17). The 307th base of the *PmGSTF2* gene in the non-green sepal-type group was G, but the 307th base of the *PmGSTF2* gene in the green sepal-type group mutated to T, causing the amino acid at this position to mutate from the original glutamate to the termination codon. We measured the anthocyanin content in young leaves of samples from different groups; the anthocyanin content in green sepal-type samples was significantly lower than that in the non-green sepal-type group (Fig. 8b). Fluorescence quantification also indicated that the expression level of the *PmGSTF2* gene in the non-green sepal-type samples was significantly higher than that in the green sepal-type group (Fig. 8c).

To further analyse the function of *PmGSTF2*, we obtained a *gst* mutant of *Arabidopsis* and back-populated the mutant with the *GST* gene missing a segment of sequence (gst/*PmGSTF2*-S) and the complete *GST* gene (gst/*PmGSTF2*-L) sequence in *P*. *mume*. The obtained transgenic strains were confirmed by β-glucuronidase (GUS) staining (Figure S18). PCR amplification of cDNA from transgenic, wild-type, and mutant samples was performed with bands of 300 bp in the gst/*PmGSTF2*-S transgenic strains and 650 bp in the gst/*PmGSTF2*-L transgenic strains, but no bands were observed in the wild-type or mutant strains (Figure S19). We seeded wild-type, mutant, gst/*PmGSTF2*-S, and gst/*PmGSTF2*-L transgenic strains simultaneously in burrow trays and subjected four-week-old seedlings to light treatments. There was no visible red colour production in the leaves of mutant and gst/*PmGSTF2*-S transgenic strains, whereas there was visible red colour production in the leaves of wild-type and gst/*PmGSTF2*-L transgenic strain seedlings (Fig. 8a). The anthocyanidin assay also indicated that the anthocyanidin content in the wild-type and gst/*PmGSTF2*-L transgenic strain samples was highly significantly higher than that in the mutant and gst/*PmGSTF2*-S transgenic strains (Fig. 8d), the expression level of *PmGSTF2* gene also showed the same result (Fig. 8e), indicating that *PmGSTF2*-L expression promoted anthocyanin accumulation, whereas *PmGSTF2*-S did not promote anthocyanin accumulation.

**Fig. 9:**
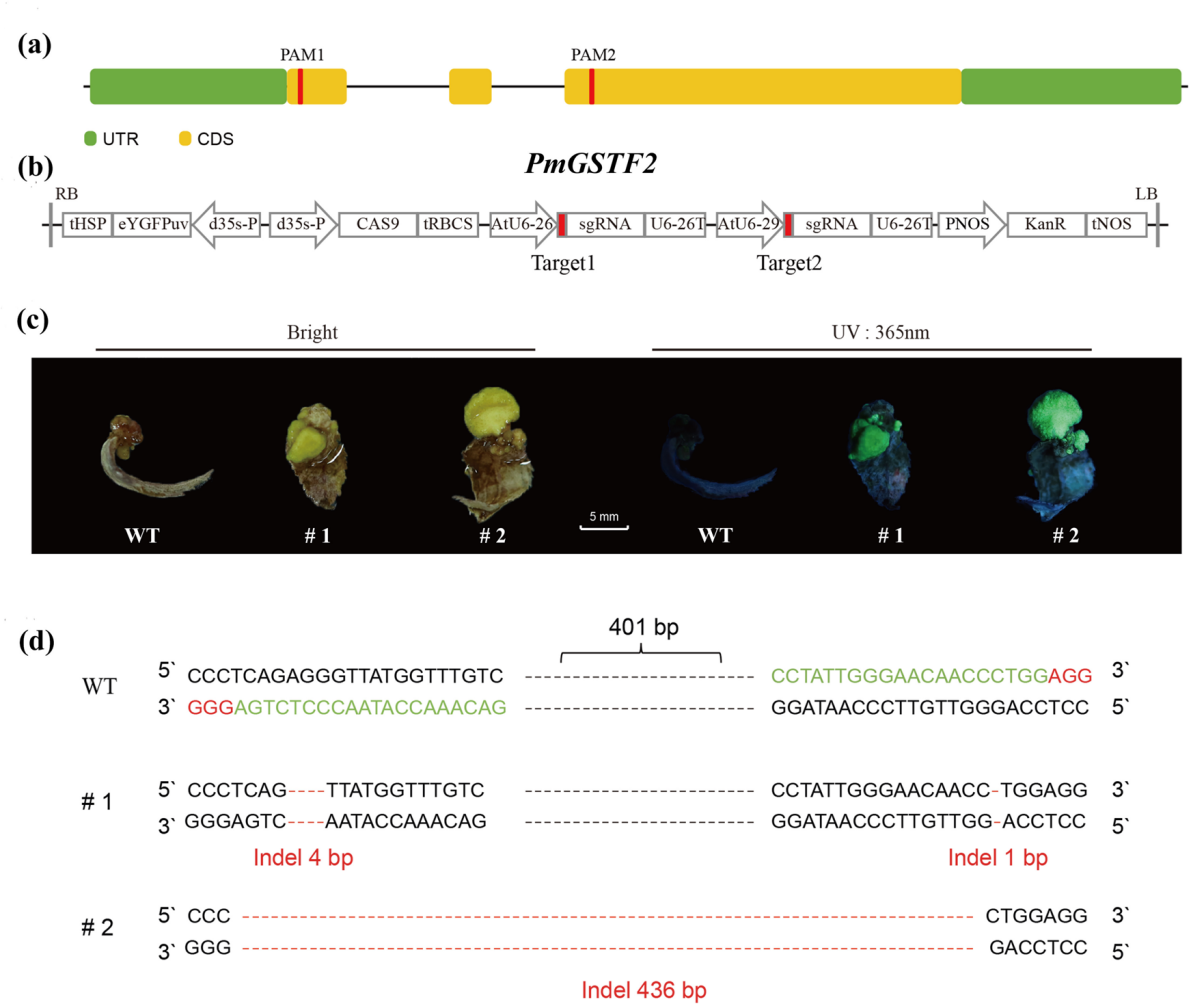
The knockout experiment of *PmGSTF2* gene. **a** The *PmGSTF2* gene structure and the PAM site; (**b**) Schematic diagram of the CRISPR/Cas9 vector; (**c**) Transgenic callus under natural light and 365 nm ultraviolet radiation; (**d**) DNA sequences of the mutated *PmGSTF2* on target1 and target2. The number of each mutation type revealed by random sequencing of cloned PCR products was used to estimate genome editing efficiency

Meanwhile, we also conducted CRISPR/Cas9 knockout experiments of *PmGSTF2* gene in the callus tissue of *Prunus mume*. Under 365 nm ultraviolet light irradiation, we observed yellow green fluorescence in the callus tissue of # 1 and # 2 leaf explants (Fig. 9c), indicating successful transfer of Cas 9. Under normal light conditions, the callus tissue (WT) of the *P. mume* Meiren we used appeared red, while the callus parts of explants # 1 and # 2 were green (Fig. 9c), indicating that knocking out the *PmGSTF2* gene using CRISPR inhibited the accumulation of anthocyanins in the callus tissue. To further verify the functional deficiency of *PmGSTF2* gene, we amplified, cloned, and sequenced the targeted region of *PmGSTF2* gene (Fig. 9d). The sequencing results showed that multiple types of mutations, including single nucleotide/dinucleotide insertions and deletions, as well as large fragment insertions and deletions, were detected within the targeted region of the designed PAM site. These mutations cause a shift in the reading frame and the production of premature stop codons, resulting in the inactivation of *PmGSTF2* gene function.

**Fig. 10:**
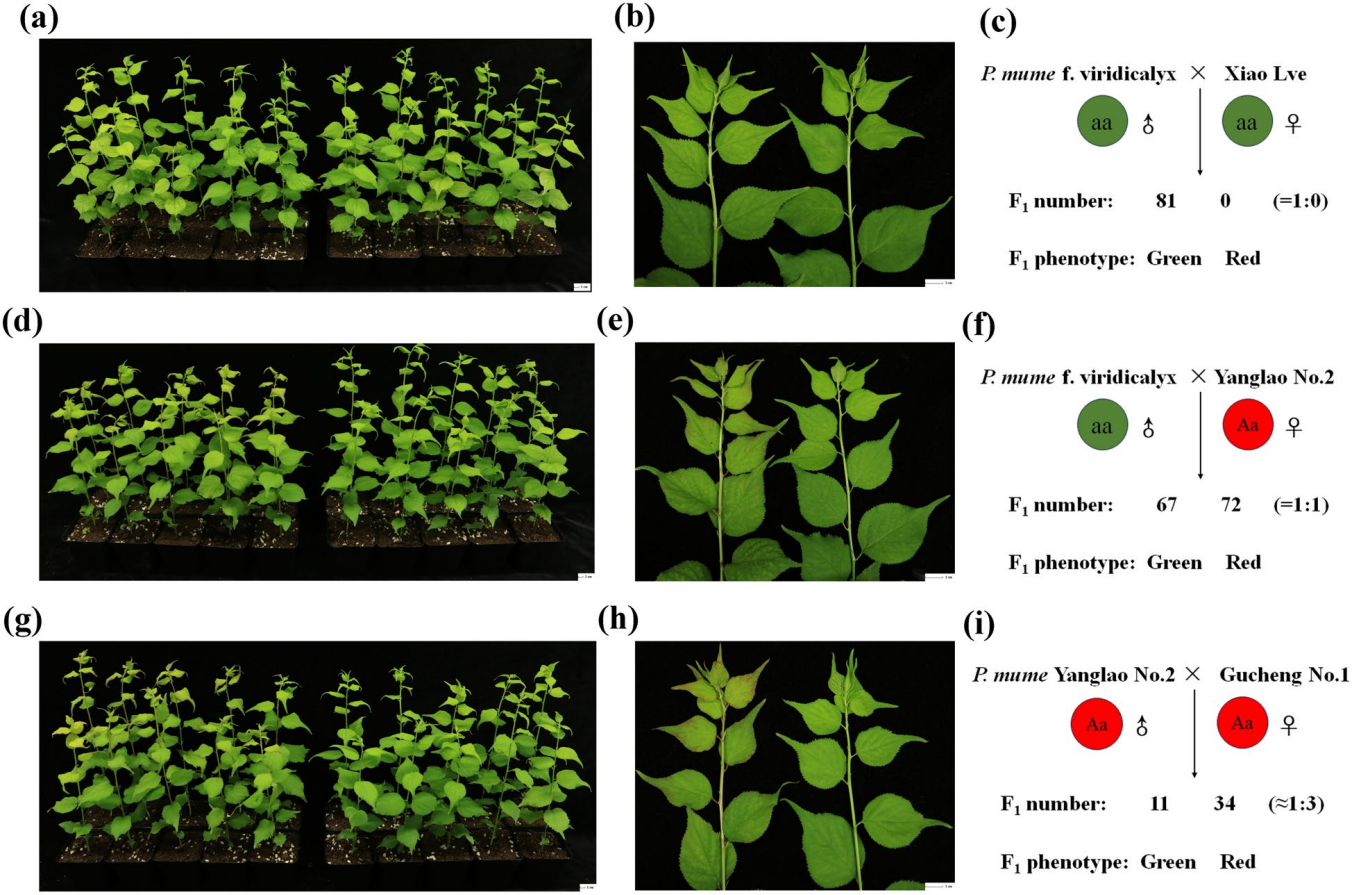
Phenotype and quantity of F_1_ seedlings in different hybrid combinations. **a** The overall phenotype of F_1_ seedlings in the hybrid combination of *P. mume* f. viridicalyx and *P. mume* Xiao Lve; (**b**) Partial phenotypic differences between F_1_ seedlings of *P. mume* f. viridicalyx and *P. mume* Xiao Lve hybrid combinations; (**c**) Schematic diagram of *P. mume* f. viridicalyx and *P. mume* Xiao Lve hybrid combinations and the number of different phenotypes in F_1_ generation; (**d**) The overall phenotype of F_1_ seedlings in the hybrid combination of *P. mume* f. viridicalyx and *P. mume* Yanglao No.2; (**e**) Partial phenotypic differences between F_1_ seedlings of *P. mume* f. viridicalyx Lve and *P. mume* Xiao Lve hybrid combinations; (**f**) Schematic diagram of *P. mume* f. viridicalyx and *P. mume* Yanglao No.2 hybrid combinations and the number of different phenotypes in F_1_ generation; (**g**) The overall phenotype of F_1_ seedlings in the hybrid combination of *P. mume* Yanglao No.2 and *P. mume* Gucheng No.1; (**h**) Partial phenotypic differences between F_1_ seedlings of *P. mume* Yanglao No.2 and *P. mume* Gucheng No.1 hybrid combinations; (**i**) Schematic diagram of *P. mume* Yanglao No.2 and *P. mume* Gucheng No.1 hybrid combinations and the number of different phenotypes in F_1_ generation

To further investigate whether the *PmGSTF2* gene mutation is co-segregated with the green sepal trait, we validated it using different hybrid combinations. The results showed that samples with homozygous *PmGSTF2* gene mutation (aa) were used as parents for hybridization (Fig. 10c), and their hybrid offspring plants were all green (Fig. 10a-b and Figure S20a). The *PmGSTF2* gene heterozygous mutation (Aa) sample was used as the maternal parent, and the *PmGSTF2* gene homozygous mutation (aa) sample was used as the paternal parent (Fig. 10f). The hybrid offspring plants were half red and half green, with a separation ratio close to 1:1 (Fig. 10d-e and Figure S20b). The samples of *PmGSTF2* gene heterozygous mutation (Aa) were used as parents for hybridization (Fig. 10i), and the hybrid offspring plants were partially green and mostly red, with a segregation ratio of nearly 1:3 (Fig. 10g-h and Figure S20c). The above research results indicate that *PmGSTF2* gene mutation is co-segregated with green sepal traits.

## Discussion

*Prunus mume*, an essential subtropical fruit tree in China, blooms early and has a relatively small genome, making it an excellent model for investigating the mechanisms underlying flowering regulation in woody plants. *Prunus mume* is used for its flowers and fruit and is a wonderful tree species for gardening and countryside construction. Assembling a highly complete, accurate, and continuous reference genome is key for genomic studies (van Rengs et al. 2022; Yang et al. 2022). Since the release of the first *P*. *mume* genome in 2012 (Zhang et al. 2012a, b), several contig-level or chromosome-level *P*. *mume* genomes have been published (Wang et al. 2021; Zheng et al. 2022). However, these genomes are incomplete and have gaps because they do not include repetitive regions, such as telomeres and centromeres, which results in the loss of a large amount of genetic information. In this study, to improve genome quality and broaden the data available for the *P*. *mume* genome, we assembled the first gap-free haplotype T2T *P*. *mume* f. viridicalyx genome by de novo assembly using ONT ultra-long, HiFi, Hi-C, and other data, which resulted in a higher quality and completeness than previously published *P*. *mume* genomes. We assembled 32 telomeres, indicating that all 16 chromosomes of the *P*. *mume* f. viridicalyx genome were assembled from telomere to telomere. We successfully predicted 16 centromere regions, which were mainly composed of repetitive sequences. This is consistent with other plant species, such as rice (Song et al. 2021), rose (Hibrand et al. 2018), banana (Belser et al. 2021), pear (Sun et al. 2023), and maize (Chen et al. 2023). The complete T2T haplotype genome will provide a reference basis for functional gene analysis and genetic evolutionary mechanism studies of *P*. *mume*.

Comparative genome analysis also showed the high completeness and quality of the *P*. *mume* f. viridicalyx genome. Phylogenetic tree analyses showed that species within the genus *Prunus*, such as *P*. *salicina*, *P*. *mume*, and *P*. *armeniaca*, form a separate branch and exhibit relatively short divergence times, which is consistent with previous findings (Huang et al. 2021; F Jiang et al. 2019; Jiu et al. 2024; Xue et al. 2019). In addition, three *P*. *mume* clustered together, and *P*. *mume* f. viridicalyx and *P. mume* var. tortuosa were more closely related to each other than to wild *P*. *mume*. The results also indicate that the ancestor of *P*. *mume* f. viridicalyx and *P. mume* var. tortuosa diverged from wild *P*. *mume* about 7.59 million years ago (Mya), and later, the ancestor of *P*. *mume* f. viridicalyx and *P. mume* var. tortuosa diverged about 4.47 million years ago, which is primarily in agreement with the results of previous studies (Tan et al. 2024; Zheng et al. 2022). The analysis of insertion time results of the LTRs also revealed that the wild *P*. *mume* genome is much more ancient, while the phylogenetic tree of the Ty3/Gypsy superfamily revealed that most LTR-RTs of wild *P*. *mume* clustered into RETAND and CRM branches, while the LTR-RTs of *P*. *mume* f. viridicalyx and *P. mume* var. tortuosa were evenly distributed among the seven clades, showing high diversity and richness, suggesting that the *P*. *mume* f. viridicalyx and *P. mume* var. tortuosa genomes had greater expansion and differentiation. The gene families also showed significant expansion and contraction of 40 and 48 gene families, respectively, in *P*. *mume* f. viridicalyx compared to those of its close relatives. Ka/Ks analysis showed that 163 genes were subjected to positive selection in LE_hap1 compared to other *P*. *mume*. Functional annotation and enrichment analysis showed that many genes involved in the stress response to different types of stimuli were significantly enriched, mainly including water deprivation, salt, viruses, DNA damage, and oxidative stress. In addition, in the KEGG enrichment pathways, significant enrichment of genes involved in carotenoid biosynthesis and amino acid metabolism was observed. A total of 143 genes were subjected to positive selection in LE_hap2 compared to other *P*. *mume*. These genes were involved in scopoletin glucosyltransferase activity, pentose and glucuronate interconversions, sulphate adenylyl transferase (ADP) activity, and terpenoid backbone biosynthesis.

*P*. *mume* f. viridicalyx has a green sepal type, belongs to the straight-branching *P*. *mume* category of the true *P*. *mume* family, and is the best of the *P*. *mume* blossom strains. It is named for its green sepals, white flowers, and green twigs and is an excellent local variety that is widely cultivated. However, the molecular mechanism underlying this anthocyanin deficiency feature is unclear. To reveal the molecular mechanism of this feature, we first explored the domestication mechanism of green sepals in *P*. *mume* through comparative genome analysis, which revealed 3796 genes with significant variation that were partially enriched in the glutathione metabolism, terpene backbone biosynthesis, carotenoid biosynthesis, and flavonoid biosynthesis pathways. We also combined population structure and selective sweep analyses to reveal that the green sepal-type samples were subjected to significant positive selection, especially on chromosome 4 with a highly selected region (1.08–1.10 Mb), which was also shown to have high linkage strength by LD linkage analyses, and one of the genes encoding a glutathione S-transferase in the green sepal-type samples with a nonsense mutation in the coding region. Glutathione transferase (GST) is an anthocyanin transporter protein that transports anthocyanins from the ER to vesicles in fruit and flowers (Y Liu et al. 2019a, b; Zhao et al. 2020). In previous studies, the function of the *GST* gene protein was lost in peach due to an early termination codon, reducing anthocyanin accumulation in peach fruit (Lu et al. 2021), whereas the *GST* in peach is homologous to the *PmGSTF2* gene in our study, with a similarity of 98%. In the active center of GST, two glutathione (GSH) ligands are crucial for catalyzing the reaction. One of the GSH ligands is mainly involved in substrate binding. This GSH ligand positions the substrate at the appropriate site of the active center, creating conditions for subsequent catalytic reactions. Another GSH ligand plays a crucial role in the catalytic process, as the thiol group of the GSH ligand can attack the carbonyl carbon, triggering the formation of covalent bonds and promoting the formation of glutathione S-conjugates between the substrate and GSH (Kampranis et al. 2000; Sandermann 1994). In this study, the codon of the *PmGSTF2* gene in *P. mume* f. viridicalyx undergoes premature termination mutation, resulting in the loss of GSH ligands (Figure S21). The overexpression experiment of *PmGSTF2*-L was shown to promote anthocyanin accumulation, whereas *PmGSTF2*-S did not, as shown by backfilling missing and complete *PmGSTF2* genes into the *Arabidopsis gst* mutation. The CRISPR/Cas9 knockout experiments of *PmGSTF2* gene in the callus tissue of *Prunus mume* also showed that knocking out the *PmGSTF2* gene using CRISPR inhibited the accumulation of anthocyanins in the callus tissue. Meanwhile, *PmGSTF2* gene mutation is co-segregated with the green sepal trait, we validated it using different hybrid combinations.

In summary, we successfully assembled a gap-free T2T haplotype genome and predicted 32 telomeres and 16 centromeres on all chromosomes, providing a high-quality reference genome and broadening the diversity of *P*. *mume* genomic resources. In addition, we further utilised genetic evolution and comparative genomic analysis of the genome and found that *PmGSTF2* plays an essential role in cyanogenic glycoside synthesis in *P*. *mume*, revealing the molecular mechanism by which nonsense mutations in *PmGSTF2* lead to green sepal features in *P*. *mume* f. viridicalyx.

## Materials and methods

### Plant materials and sequencing

The fresh and young leaves of *P. mume f. viridicalyx* at an early-to-mid developmental stage were collected and frozen in liquid nitrogen in the National GenBank of *Prunus mume* in China. Total genomic DNA was extracted using a Plant Genomic DNA Kit from TIANGEN (DP305-03; Tiangen, Beijing, China). High-throughput sequencing was performed using the Illumina NovaSeq 6000 series sequencer (PE150). Fastp software was used to filter raw data, and high-quality reads obtained after quality control were called clean data.

The Nanopore genome sequencing process was used to construct the DNA library after the samples passed the quality test. The DNA library of a certain concentration and volume was added to the flow cell and then sequenced using an Oxford Nanopore PromethION sequencer. The obtained data were filtered using Filtlong software (https://github.com/rrwick/Filtlong), while data with an N50 of not less than 100 kb were extracted for subsequent assembly.

The Pacbio genome sequencing process was used to fragment the DNA after the samples were qualified by quality control. The fragmented DNA sequences were repaired, and spliced sequences were ligated. Data underwent enzymatic processing and purification and were sequenced using the HiFi sequencing mode of the PacBio SequeII platform. The HiFi data were assembled using the default parameters of Hifiasm software (Cheng et al. 2021), and the main contigs obtained were adjusted for subsequent analysis.

Fresh tissues, including flower, fruit, root, stem, and leaf tissues, were collected from the same *P. mume* f. viridicalyx tree and immediately frozen in liquid nitrogen. We used a Plant Genome RNA Kit from TIANGEN to extract the total RNA according to the manufacturer’s instructions. Second-generation mRNA sequencing was performed on the Illumina Novaseq X Plus Platform. The Nanopore transcriptome sequencing process was performed on an Oxford Nanopore PromethION Sequencer.

### Genome de novo assembly and quality assessment

Genomic assembly was performed using NECAT and Hifiasm software (Chen et al. 2021). Two rounds of consensus correction were performed using Racon (Vaser et al. 2017) with corrected PacBio long reads, and the resulting assembly was further polished using Pilon (Walker et al. 2014) with default parameters based on Illumina sequencing data. We used juicer (v2.0, Durand et al. 2016) and 3D-DNA software (Dudchenko et al. 2017) to cluster the contigs assembled by Hifiasm software and determine the correlation between contigs. Then, Juciertools software was used to convert the interaction relationships between contigs into specified binary files and manually correct the sequenced and oriented contigs using Juciebox software (v2.15.07, Robinson et al. 2018) to obtain the final assembly results at the chromosome level. For chromosomes with gaps or missing telomeres, Minimap2 software (Li 2018) was used to align HiFi and ultra-long data to the vicinity of telomeres for further manual adjustment.

After assembly, different approaches were employed to evaluate genome quality. First, the Illumina reads for the genome survey were mapped to the final assembled genome using BWA software (Li and Durbin 2009). For chromosomes with gap or missing telomeres, HiFi and ultra-long data were aligned near the telomeres using Minimap2 software (Li 2018) and further adjusted manually. BUSCO software (Simão et al. 2015) was used to evaluate the genome assembly and annotation quality based on homology alignment, and BUSCO ratings were generally greater than 90%, which was considered a good genome annotation. We used LTR-FINDER-Parallel and LTRharvest to predict and identify LTRs and then calculated LAI values using LTR-Retriever to evaluate the continuity of genome assembly (Ou et al. 2018). The consensus quality value (QV) used to assess genomic accuracy was determined with Merqury software (Rhie et al. 2020); a higher QV value represents a higher accuracy.

### Repeat sequence annotation

Repeat sequence de novo prediction of the genome was first performed using RepeatModeler (Flynn et al. 2020), and the predicted results were merged with the RepBase database. Repeat sequence prediction of the genome was performed using RepeatMasker (Tarailo-Graovac and Chen 2009). Repeat sequences were predicted using the RepeatProteinMask tool in RepeatMasker, and the two sequences were integrated.

### Gene prediction and functional annotation

mRNA prediction was performed using three methods: de novo prediction, homology prediction, and transcript prediction. De novo prediction was performed using GlimmerHMM v3.0.4 software (Majoros et al. 2004) and Augustus (Nachtweide and Stanke 2019), and homologous prediction was performed using GeMoMa v1.9 software (Keilwagen et al. 2019) with species, including *P*. *mira*, *P*. *ducis*, *P*. *avium*, and *P*. *mume*. RNA sequencing (RNA-seq) data were reconstructed from transcripts obtained using StringTie (Pertea et al. 2015), followed by the use of the TransDecoder (Grabherr et al. 2011) to predict coding frames. We used EVidenceModeler software to integrate multiple datasets (Haas et al. 2008), and the integrated data were updated using PASA (Haas et al. 2003) to add UTR regions and discover new transcripts.

The nucleic acid sequences of the longest transcripts of the predicted genes were compared using the clusters of orthologous groups (COG), non-redundant (NR), clusters of orthologous groups for eukaryotes (KOG), GO, Swiss-Prot, and KEGG databases using BLAST software (Altschul et al. 1990) and HMMER v3.2.1 (Eddy 2009) to compare amino acid sequences with the Pfam database and annotate predicted genes.

### Non-coding RNA annotation

Non-coding RNA prediction consisted of three parts: rRNA sequence prediction using barrnap (N Liu et al. 2019a, b); and tRNASCAN v2.0.0 (Chan et al. 2021) to predict tRNA sequences. INFERNALv1.1.2 (Nawrocki and Eddy 2013) was used to search for ncRNA sequences, such as snRNA and miRNA, in the genome by searching the Rfam database.

### Genome synteny analysis and comparative analysis

All proteins of *P*. *mume* f. viridicalyx, wild *P*. *mume*, and *P*. *mume* var. tortuosa were aligned using BLASTP software, and MCscanX software (Wang et al. 2012) was used to identify the syntenic regions between species based on the BLASTP results. In addition, all protein sequences from five subgenus *Prunus* species, including *P*. *mume*, *P*. *mume* Tortuosa, *P*. *armeniaca*, and *P*. *salicina*, were used to generate gene family clusters.

### Gene family identification and species phylogenetic tree construction

Genome files and annotation files of *P*. *mume* f. viridicalyx and 11 other species (*P*. *persica*, wild *P*. *mume*, *P*. *mume* var. tortuosa, *P*. *davidiana*, *P*. *dulcis*, *P*. *armeniaca*, *P*. *mira*, *P*. *salicina*, *P*. *kanzakura*,* P*. *yedoensis*, and *A*. *thaliana*) were prepared, and the longest amino acid sequences were extracted for analysis. The homology of all sample genes was identified using OrthoFinder software (Emms and Kelly 2019) to identify gene families and clustered using Diamond v2.0.6.144 software. The single-copy genes in the OrthoFinder results were aligned head-to-tail using MAFFT (v7.427) and subjected to evolutionary tree analysis using RAxML software (Stamatakis 2014) to obtain a maximum likelihood evolutionary tree.

### Analysis of the expansion and contraction of gene families and estimation of divergence time

The MCMCtree programme implemented in PAML was used to calculate the plant species divergence time (Yang 2007), and we obtained two calibration points on the TimeTree website to calibrate the divergence time. The divergence times between *P*. *mume* and *P*. *persica* ranged from 3.8 to 54.3 Mya, while those between *P*. *kanzakura* and* P*. *yedoensis* ranged from 33.7 to 61.5 Mya. Gene family expansion and contraction analyses were performed using CAFE v4.2.1 software (De Bie et al. 2006). Families with gene counts greater than 100 were removed from the analysis.

### Positive selection and whole-genome duplication analysis

Amino acid sequence comparison of paired genes in the MCScanX results was performed using MUSCLE v3.8.31 and ParaAT software (Zhang et al. 2012a, b) to convert amino acid sequences to nucleotide sequences. KaKs_Calculator v2.0 software (Wang et al. 2010) was used to calculate the Ka/Ks values of the genes, and the calculation method was selected as YN. Synonymous mutations are not subject to natural selection. Mutations at codon-synonymous sites are completely random and accumulate over time. If a species undergoes a genome-wide doubling event, a certain number of genes remain in the existing genome, and these genes are close to each other in terms of the synonymous mutations accumulated throughout evolution. Therefore, the calculated Ks values will be close to each other. In addition, if any nucleotide change at a site of the codon does not affect the amino acid it encodes, the site is called a 4DTV, which was calculated as described above.

### Resequencing, population structure, and selective sweep analysis

Fifteen cultivars of the *P*. *mume* f. viridicalyx from the National Genebank of *Prunus mume* in China were selected for resequencing analysis, and the genomic DNA was extracted from the fresh and young leaves as described above. Sequencing was performed using Illumina NovaSeq 6000 PE sequencing according to the manufacturer’s protocol, with at least 30 × average coverage for each accession. All reads were pre-processed, as mentioned above, for quality. The resequencing data of 15 cultivated green sepal samples, and 59 previously cultivated and wild samples (Table S25) were mapped to the wild *P*. *mume* reference genome using BWA (v.0.7.17) (Li and Durbin 2009). The mapped reads were sorted according to their genomic coordinates using Samtools (Li et al. 2009). Picard software was used to remove the duplicates, and HaplotypeCaller from GATK (McKenna et al. 2010) was used to call individual-specific gvcf files. Further STRUCTURE analyses were performed to detect subpopulations for each group by setting K from 2 to 4. For selective sweep analysis, two methods were used to detect the regions under selection (Huang et al. 2022). All candidate regions were annotated using BLASTx in the COG, NR, GO, Swiss-Prot, and KEGG databases.

### DNA verification and cloning of the *PmGSTF2* gene from *Prunus mume*

Genomic DNA was extracted from young *P*. *mume* leaves using a plant DNA extraction kit, and *PmGSTF2* primers were designed for DNA verification (Table S26). An RNA extraction kit was used to extract total RNA and reverse transcription as a template for gene cloning. Primers *PmGSTF2*-L and *PmGSTF2*-S were designed for gene cloning (Table S26). PCR products were recovered using a gel recovery kit and ligated into a cloning vector. The PCR products were transformed into *Escherichia coli* DH5α competent cells, and single colonies were selected for PCR detection. Plasmid DNA was extracted for sequencing (General Biol, Anhui, China). The SYBR PreMix Ex Taq kit (TaKaRa) was used to conduct qRT-PCR on a Bio-Rad IQ5 fluorescent quantitative PCR platform. The relative gene expression levels were calculated using the 2^−ΔΔCT^ method (Pfaffl 2001).

### Construction of the expression vector and transformation of the *PmGSTF2* gene

Based on the expression vector sequence and the *PmGSTF2*-L and *PmGSTF2*-S gene sequences, we designed specific recombinant primers PmGSTF2-L-pCAMBIA1301 and PmGSTF2-S-pCAMBIA1301 (Table S26) to amplify the *PmGSTF2*-L and *PmGSTF2*-S genes and recombine them into the pCAMBIA1301 vector. Recombinant vectors pCAMBIA1301-PmGSTF2-L and pCAMBIA1301-PmGSTF2-S were obtained and sent to the company for sequencing and plasmid extraction. The correct recombinant plasmids pCAMBIA1301-PmGSTF2-L and pCAMBIA1301-PmGSTF2-S were introduced into *Agrobacterium tumefaciens* by the heat shock method, and *Arabidopsis thaliana* was transformed using the dipping flower method (Zhang et al. 2006). The transgenic plants were screened on 1/2 Murashige and Skoog (MS) medium containing hygromycin. GUS staining was used to identify the positive strains. Specific primers were used to detect the expression levels of *PmGSTF2*-L and *PmGSTF2*-S in the positive transgenic lines.

### Construction of CRISPR system

Whole genome scanning of PAM sites with default parameters was performed using CRISPR-offinder (v1.2). Select two PAM sites with high editing efficiency and low off target activity as target sequences (Zhao et al. 2017) (Table S27). The two single-guide RNA (sgRNA) were driven separately by AtU6-29 and AtU9-26, respectively, and introduced into the CRISPR/Cas9 vector through homologous recombination. We designed two specific sgRNAs based on the genome sequence, targeting the first and third exons of the *PmGSTF2* gene, respectively (Fig. 9a). The selected target sequences (5'-CCTATTGGGAACAACCCTGG-3'and 5'-GACAAACCATAACCCTCTGA-3') exhibit high specificity and no detectable off-target sites (Fig. 9b). By assembling PAM sites and sgRNA, sequencing was performed to confirm and introduce them into Agrobacterium cells for transformation. The fluorescence of the transformed callus was detected, and used a 365 nm ultraviolet flashlight to irradiate the callus tissue under dark conditions. The callus explants transferred into the carrier will appear fluorescent green.

### Determination of anthocyanin content

The young fresh leaves of ‘Green sepal’ and ‘non-Green sepal’ were selected. Samples weighing 1.000 g were ground in liquid nitrogen, and an appropriate amount of ethanol containing 1% hydrochloric acid was added. A 10-mL volume was transferred to a container and extracted in a shaking bed (100 r/min) at 4℃ for 2 h. The extracts were filtered and set aside. A 2-mL volume of the sample solution was mixed with pH 1.0 KCI-HCI buffer (pH 4.5) and CH3COONa-HCl buffer diluted to 5 mL. After mixing, absorbance was measured at 510/700 nm, and the anthocyanin content of anthocyanin was calculated. The anthocyanin content was determined in 4-week-old wild-type, mutant, and transgenic lines of *Arabidopsis*, following the previously described methods.

## Supplementary Information

Additional file 1: Table S1. Three-generation sequencing statistics. Table S2. Assembly results based on HiFi data. Table S3. Second-generation sequencing statistics. Table S4. Assembly results based on Hi-C data. Table S5. Telomere sequence length statistics. Table S6. Length statistics of centromere sequence on chromosomes. Table S7. Genomic quality assessment based on Benchmarking Universal Single-Copy Orthologs (BUSCO) results. Table S8. The LTR Assembly Index (LAI) value of Prunus mume ‘LE_hap1’ genome. Table S9. The LTR Assembly Index (LAI) value of Prunus mume ‘LE_hap2’ genome. Table S10. The consensus quality value of Prunus mume ‘LE_hap1’ genome. Table S11. The consensus quality value of Prunus mume ‘LE_hap2’ genome. Table S12 Statistics of mRNA annotation results. Table S13. Statistics of non-coding RNA annotation results. Table S14. Repeated sequence prediction results statistics. Table S15. The specific genes were identified in the ‘LE_hap1’ genome. Table S16. The specific genes were identified in the ‘LE_hap2’ genome. Table S17. The specific genes were identified in the wild genome. Table S18. The number and percent of SNP and InDel in different regions. Table S19. The number and percent of different types of SNP and InDel. Table S20. Thestructural annotations and sequence annotations based on comparative genome analysis between wild and ‘LE_hap1’ genomes. Table S21. The structural annotations and sequence annotations based on comparative genome analysis between wild and ‘LE_hap2’ genomes. Table S22. The structural annotations and sequence annotations based on comparative genome analysis between ‘LE_hap1’ and ‘LE_hap2’ genomes. Table S23. The number and percent of SV in different regions. Table S24. The number and percent of different types of SV. Table S25. Statistics of Prunus mume genome resequencing for each accession. Table S26. Primer sequence used in this study. Table S27. Screening of PAM site in the Prunus mume f. viridicalyx whole genome using CRISPR offinder software.

Additional file 2: Figure S1. Gene comparison map of six closely related species. The horizontal axis of the four graphs represents the length of each gene (a), CDS length (b), exon length (c), and intron length (d) (window=30 bp); The vertical axis of the four graphs represents the percentage of genes of a certain statistical length to the total number of genes. Different colored lines represent different species. A total of six genomes, including Malus × domestica, Prunus avium, Prunus dulcis, Prunus mira, Prunus mume and Pyrus betulifolia. Figure S2. The upset diagram represents the shared and unique gene families among five closely related plants in the genus Prunus (P. armeniaca, P. salicina, P. mume ‘LE_hap1’ and ‘LE_hap2’, wild P. mume, and P. mume Tortuosa). Each number represents the number of gene families. Figure S3. The GO functional annotation of unique genes in Prunus mume LE. The horizontal axis represents the functions of different annotations, mainly divided into three categories: cellular components, molecular functions, and biological processes, the vertical axis represents the number of genes. Figure S4. The KEGG functional annotation of unique genes in Prunus mume LE. The size of the circle represents different numbers of genes, and the color of the circle represents different qvalues. Figure S5. The number of different types of transposons in four Prunus mume (P. mume ‘LE_hap1’, P. mume ‘LE_hap2’, wild P. mume and P. mume Tortuosa) genomes. The horizontal axis represents different types of transposons, and the vertical axis represents the specific number of transposons. Different colors represent different types of transposons. Figure S6. The percent of different types of transposons in four Prunus mume (P. mume ‘LE_hap1’, P. mume ‘LE_hap2’, wild P. mume and P. mume Tortuosa) genomes. The horizontal axis represents different types of transposons, and the vertical axis represents the proportion of transposons in the genome. Different colors represent different types of transposons. Figure S7. The size of different types of transposons in four Prunus mume (P. mume ‘LE_hap1’, P. mume ‘LE_hap2’, wild P. mume and P. mume Tortuosa) genomes. The horizontal axis represents different types of transposons, and the vertical axis represents the total length of transposons. Different colors represent different types of transposons. Figure S8. Distribution of Gypsy and Copia type LTRs along chromosomes in the four Prunus mume genomes. (a) Prunus mume LE_hap1; (b) Prunus mume LE_hap2; (c) Prunus mume Tortuosa; (d) wild Prunus mume. The horizontal axis represents the 8 chromosomes of the Prunus mume genome, and the vertical axis represents the number of LTR transposons. Figure S9. The analysis of whole genome duplication. (a) Horizontal coordinate indicates Ks, vertical coordinate indicates share (%); (b) Horizontal coordinate indicates 4DTV value, vertical coordinate indicates share (%). A total of 10 genomes and Prunus mume LE_hap1 were analyzed, including Arabidopsis thaliana, Prunus armeniaca, Prunus davidiana, Prunus dulcis, Prunus kanzakura, Prunus mira, Prunus salicina, Prunus yedoensis, Prunus persica, Prunus mume Tortuosa, Prunus mume LE_hap2. Figure S10. Chromosome-level collinearity patterns among Prunus mume species. The numbers indicate the pseudochromosome order generated from the original genome sequence. Each rounded rectangle represents a chromosome. Each color line represents 1 block. One block means that more than five paired genes were aligned in sequence. Figure S11. Positive selection of genes and enrichment analysis. (a)Analysis of non-synonymous mutation rate (Ka) and synonymous mutation rate (Ks) values. Horizontal coordinates indicate Ka/Ks, vertical coordinates indicate percentage (%), and lines of different colors indicate comparisons between species. (b) Venn plots of genes with Ka/Ks>1 in the P. mume LE_hap1 with wild P. mume and P. mume Tortuosa genomes and genes with Ka/Ks>1 in the P. mume LE_hap2 with wild P. mume and P. mume Tortuosa genomes. (c) GO and KEGG functional enrichment of the 163 shared genes in the P. mume LE_hap1 with wild P. mume and P. mume Tortuosa genomes. (d) GO and KEGG functional enrichment of the 143 shared genes in the P. mume LE_hap2 with wild P. mume and P. mume Tortuosa genomes. The size of the circle represents different numbers of genes, and the color of the circle represents different qvalues. Figure S12. The GO (a) and KEGG (b) pathway analysis of specific genes in the Prunus mume LE_hap1 genome. The size of the circle represents different numbers of genes, and the color of the circle represents different qvalues. Figure S13. The GO (a) and KEGG (b) pathway analysis of specific genes in the Prunus mume LE_hap2 genome. The size of the circle represents different numbers of genes, and the color of the circle represents different qvalues. Figure S14. The specific genes in the wild Prunus mume genome, compared to the Prunus mume LE_hap1 and LE_hap2 genomes. Figure S15. The GO (a) and KEGG (b) pathway analysis of specific genes in the wild Prunus mume genome. The size of the circle represents different numbers of genes, and the color of the circle represents different qvalues. Figure S16. Analysis of regions and genes under selection of populations at different phenotypes. The distribution of FST (a) and Pi (b) values for the selective sweep analysis in different phenotypes groups, the position of the dashed line represents the top 5% of the selected areas. (c) Venn diagram showing the number of genes under selection in the two groups and comparative genomic analysis. Over-represented Gene Ontology (d) terms and Kyoto Encyclopedia of Genes and Genomes (e) pathways in overall selection. The size of the circle represents different numbers of genes, and the color of the circle represents different qvalues. Figure S17 The nucleotide sequence alignment of PmGSTF2 gene in samples with low and high anthocyanin content groups. The sequence TH3H, YLQ, XYQ and YDM represent the samples in the non-green sepal type group, sequence LE, LM, XLE and JQLE represent the samples in the green sepal type group, and the green box represents the mutation position. LE: Lv E; LM: Lv mei; XLE: Xiao Lve; JQLE: Jinqian Lve; TH3H: Taihu No.3; YDM: Yadan Mei; XYQ: Xiye Qing; YLQ: Yeli Qing. Figure S18. The GUS staining of wild-type, mutant, and transgenic strains of Arabidopsis thaliana. Figure S19. The PCR amplification of transgenic, mutant, and wild-Type Arabidopsis cDNAs validates missing and complete PmGSTF2 genes. Figure S20. The peak plot of sequencing results of PmGSTF2 gene in F1 seedlings of different hybrid combinations. (a) F1 seedlings in the hybrid combination of P. mume f. viridicalyx and P. mume Xiao Lve, the nonsense mutation sites of PmGSTF2 gene in all green phenotype samples exhibit a single peak of A base; (b) F1 seedlings in the hybrid combination of P. mume f. viridicalyx and P. mume Yanglao No.2, the nonsense mutation sites of PmGSTF2 gene in all green phenotype samples exhibit a single peak of A base, and in all red samples exhibit A/C base heterozygosity peak; (c) F1 seedlings in the hybrid combination of P. mume Yanglao No.2 and P. mume Gucheng No.1, the nonsense mutation sites of PmGSTF2 gene in all green phenotype samples exhibit a single peak of A base; Some of the nonsense mutation sites in the PmGSTF2 gene in the red phenotype samples exhibit a single peak at the C base, while others exhibit a heterozygous peak at the A/C base. The red box represents the site of nonsense mutation in PmGSTF2 gene. Figure S21. The three-dimensional structure of PmGSTF2 gene protein. (a) The structure of PmGSTF2-L; (b) The structure of PmGSTF2-S.

## Data Availability

All data supporting the results of this study are included in the manuscript and its additional files. The genome sequencing data have been deposited in the NCBI database under accession number is PRJNA1127312. The genome assemblies and annotation have been deposited in the Figshare database (DOI: 10.6084/m9.figshare.26105437).

## Abbreviations

4DTV: Fourfold degenerate synonymous site
BUSCO: Benchmarking Universal Single-Copy Orthologs
COG: Clusters of orthologous groups database
GO: Gene Ontology
KEGG: Kyto Encyclopaedia of Genes and Genomes
KOG: Clusters of orthologous groups for eukaryotes database
LTR: Long terminal repeat
NR: Non-redundant database
OE: Overexpression
ONT: Oxford Nanopore Technologies
PCA: Principal component analysis
qRT-PCR: Quantitative real-time polymerase chain reaction
QV: Consensus quality value
SNP: Single nucleotide polymorphisms
T2T: Telomere-to-telomere
TE: Transposon elements
WT: Wild-type
