# Supplementary material for: The telomere-to-telomere haplotype genome provides in-depth insights into the molecular mechanisms of the anthocyanin deficiency phenotype in Prunus mume
Source: Mol Hortic. 2026 Feb 2;6:8. doi: 10.1186/s43897-025-00186-8 (PMC12862912; doi:10.1186/s43897-025-00186-8)
Supplement: Supplementary file 2 — Additional file 2: Figure S1. Gene comparison map of six closely related species. The horizontal axis of the four graphs represents the length of each gene (a), CDS length (b), exon length (c), and intron length (d) (window=30 bp); The vertical axis of the four graphs represents the percentage of genes of a certain statistical length to the total number of genes. Different colored lines represent different species. A total of six genomes, including Malus × domestica, Prunus avium, Prunus dulcis, Prunus mira, Prunus mume and Pyrus betulifolia. Figure S2. The upset diagram represents the shared and unique gene families among five closely related plants in the genus Prunus (P. armeniaca, P. salicina, P. mume ‘LE_hap1’ and ‘LE_hap2’, wild P. mume, and P. mume Tortuosa). Each number represents the number of gene families. Figure S3. The GO functional annotation of unique genes in Prunus mume LE. The horizontal axis represents the functions of different annotations, mainly divided into three categories: cellular components, molecular functions, and biological processes, the vertical axis represents the number of genes. Figure S4. The KEGG functional annotation of unique genes in Prunus mume LE. The size of the circle represents different numbers of genes, and the color of the circle represents different qvalues. Figure S5. The number of different types of transposons in four Prunus mume (P. mume ‘LE_hap1’, P. mume ‘LE_hap2’, wild P. mume and P. mume Tortuosa) genomes. The horizontal axis represents different types of transposons, and the vertical axis represents the specific number of transposons. Different colors represent different types of transposons. Figure S6. The percent of different types of transposons in four Prunus mume (P. mume ‘LE_hap1’, P. mume ‘LE_hap2’, wild P. mume and P. mume Tortuosa) genomes. The horizontal axis represents different types of transposons, and the vertical axis represents the proportion of transposons in the genome. Different colors repr [file 43897_2025_186_MOESM2_ESM.docx]

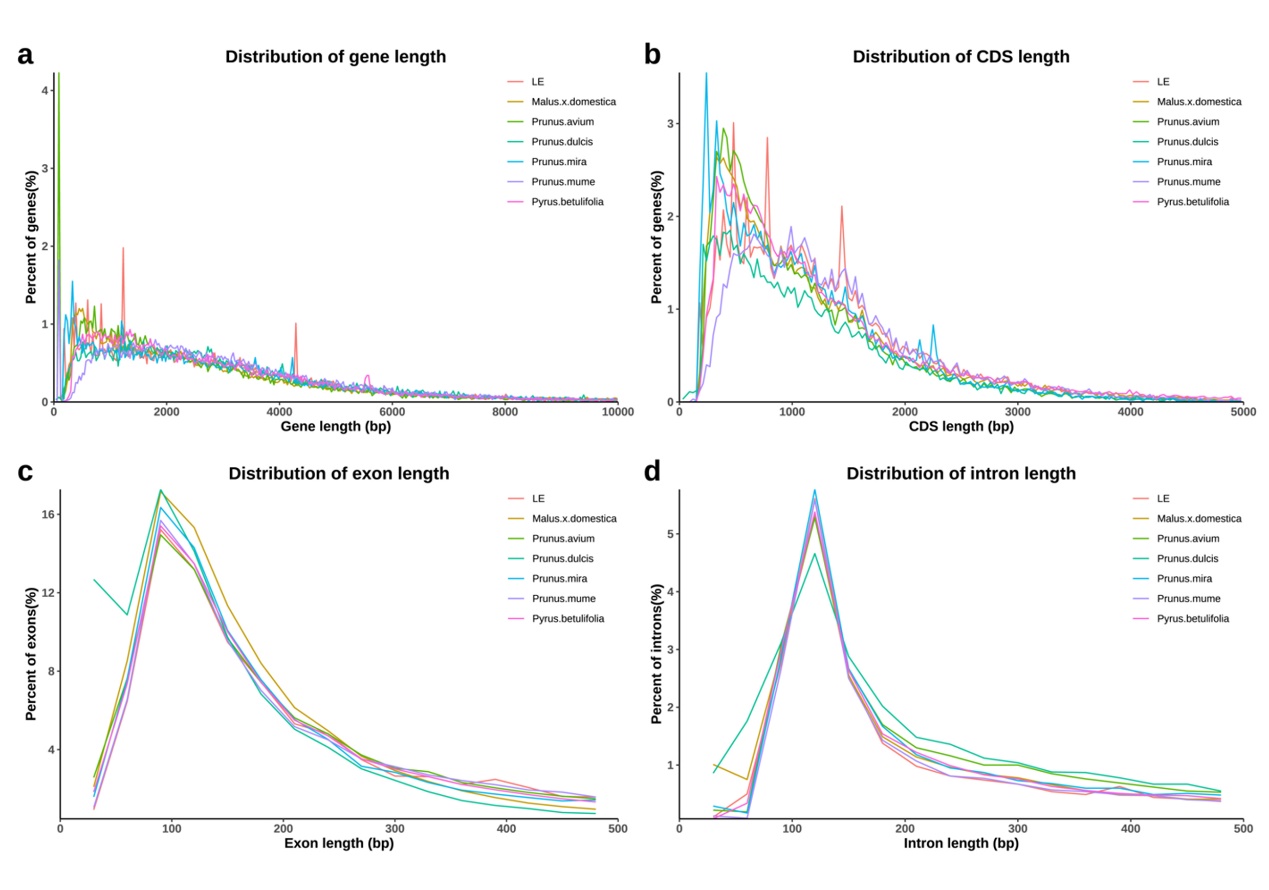


**Figure S1 Gene comparison map of six closely related species.** The horizontal axis of the four graphs represents the length of each gene (a), CDS length (b), exon length (c), and intron length (d) (window=30 bp); The vertical axis of the four graphs represents the percentage of genes of a certain statistical length to the total number of genes. Different colored lines represent different species. A total of six genomes, including *Malus × domestica*, *Prunus avium*, *Prunus dulcis*, *Prunus mira*, *Prunus mume* and *Pyrus betulifolia*.


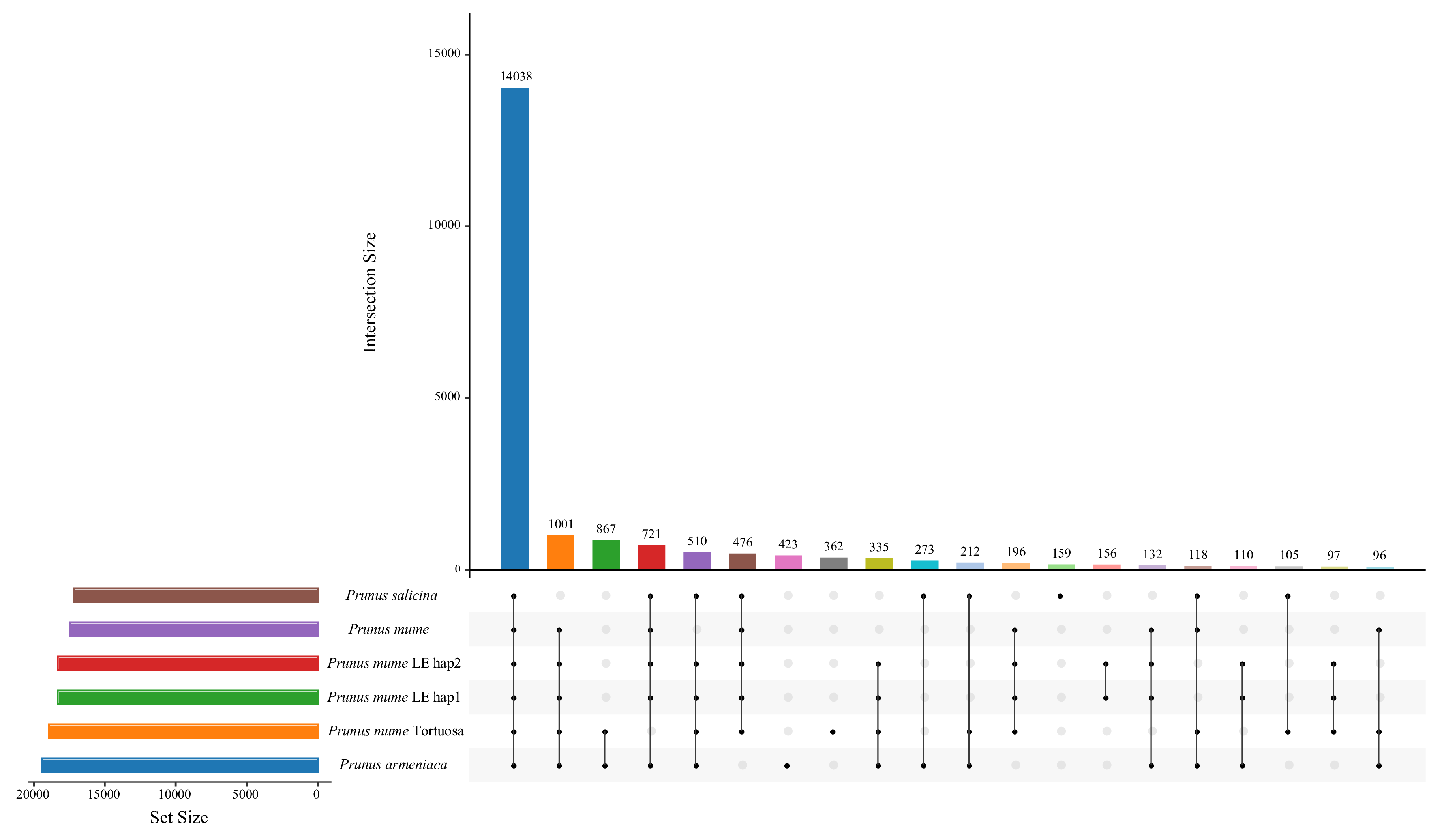


**Figure S2 The upset diagram represents the shared and unique gene families among five closely related plants in the genus Prunus (*P. armeniaca*, *P. salicina*, *P. mume* LE_hap1 and LE_hap2, wild *P. mume*, and *P. mume* Tortuosa).** Each number represents the number of gene families


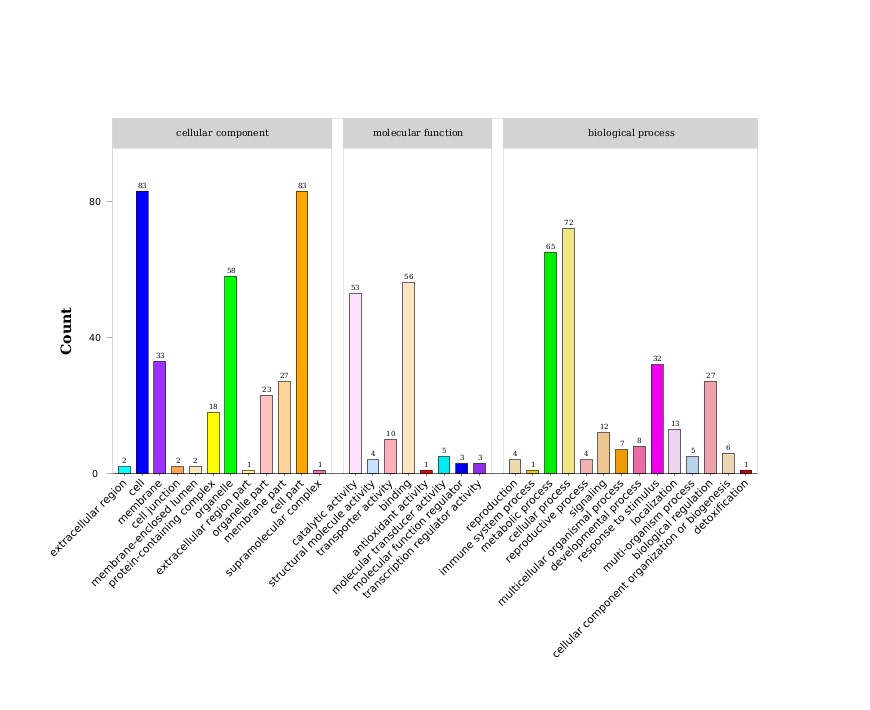


**Figure S3 The GO functional annotation of unique genes in *Prunus mume* LE.** The horizontal axis represents the functions of different annotations, mainly divided into three categories: cellular components, molecular functions, and biological processes, the vertical axis represents the number of genes.


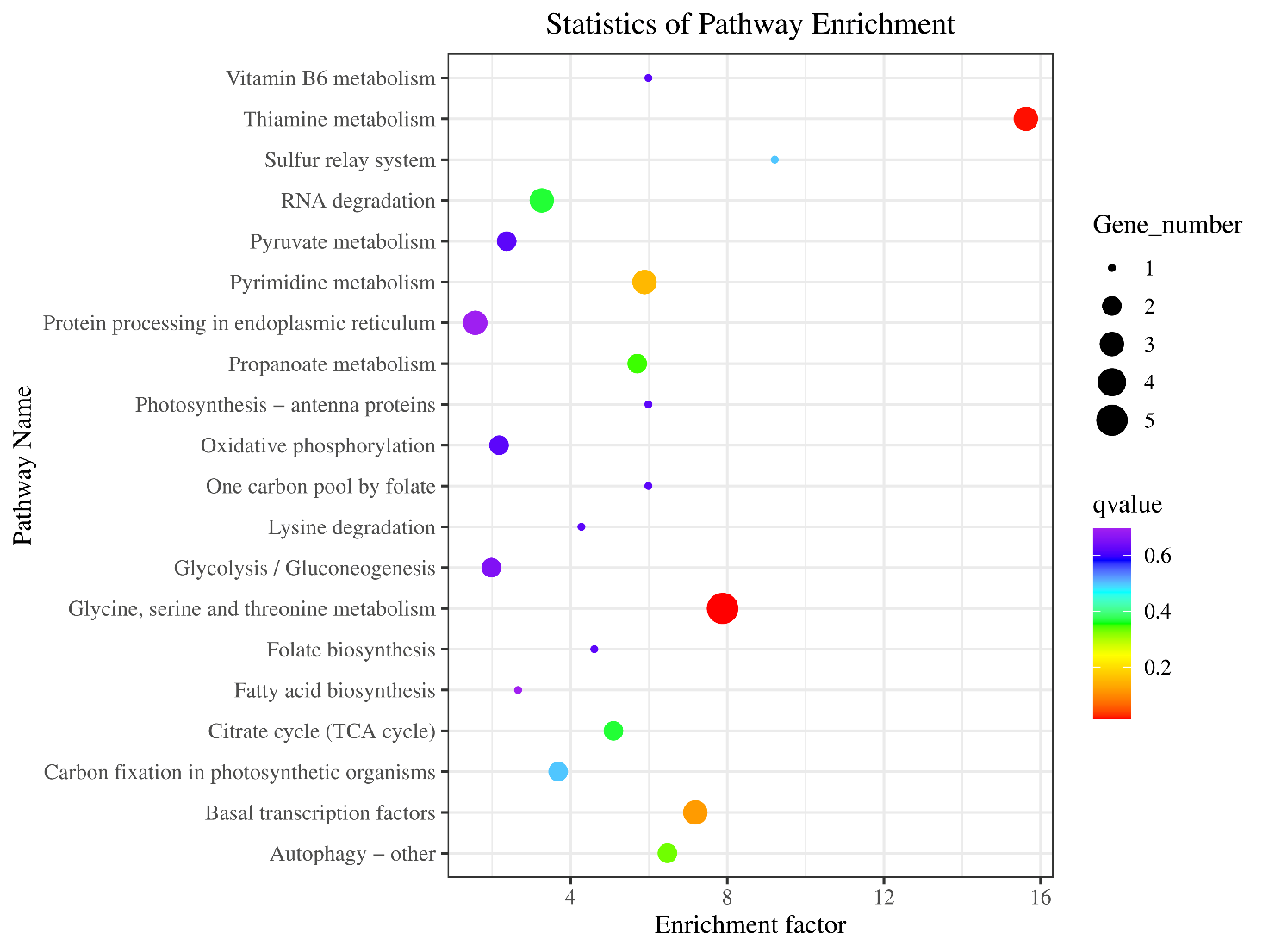


**Figure S4 The KEGG functional annotation of unique genes in *Prunus mume* LE.** The size of the circle represents different numbers of genes, and the color of the circle represents different qvalues.


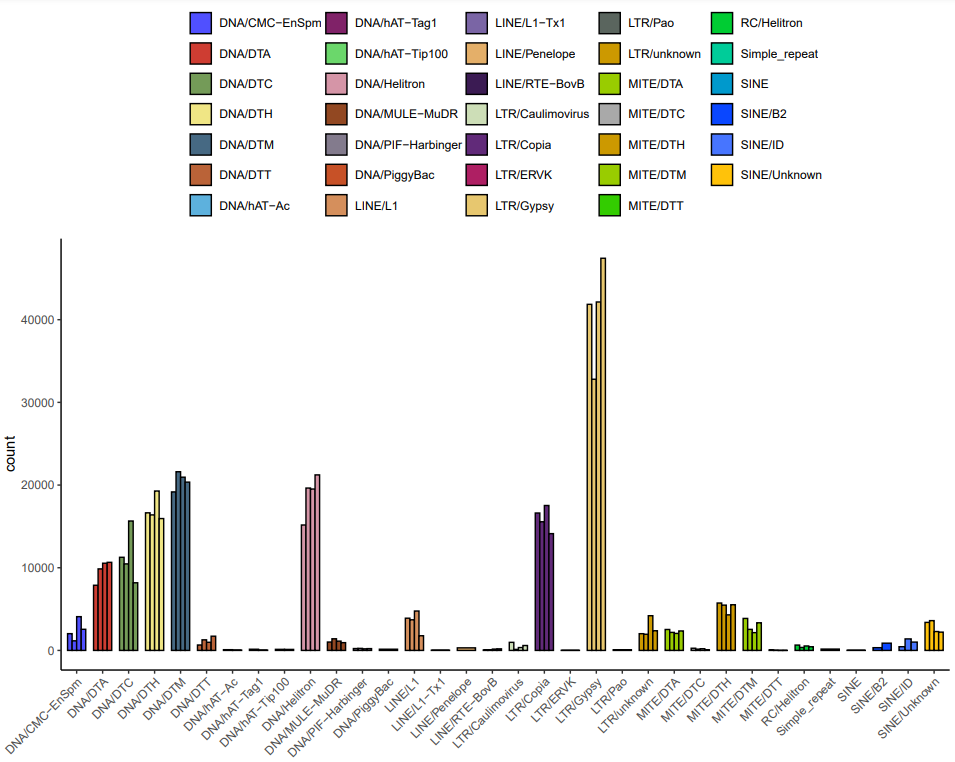


**Figure S5 The number of different types of transposons in four *Prunus mume* (*P. mume* ‘LE_hap1’, *P. mume* ‘LE_hap2’, wild *P. mume* and *P. mume* Tortuosa) genomes.** The horizontal axis represents different types of transposons, and the vertical axis represents the specific number of transposons. Different colors represent different types of transposons.


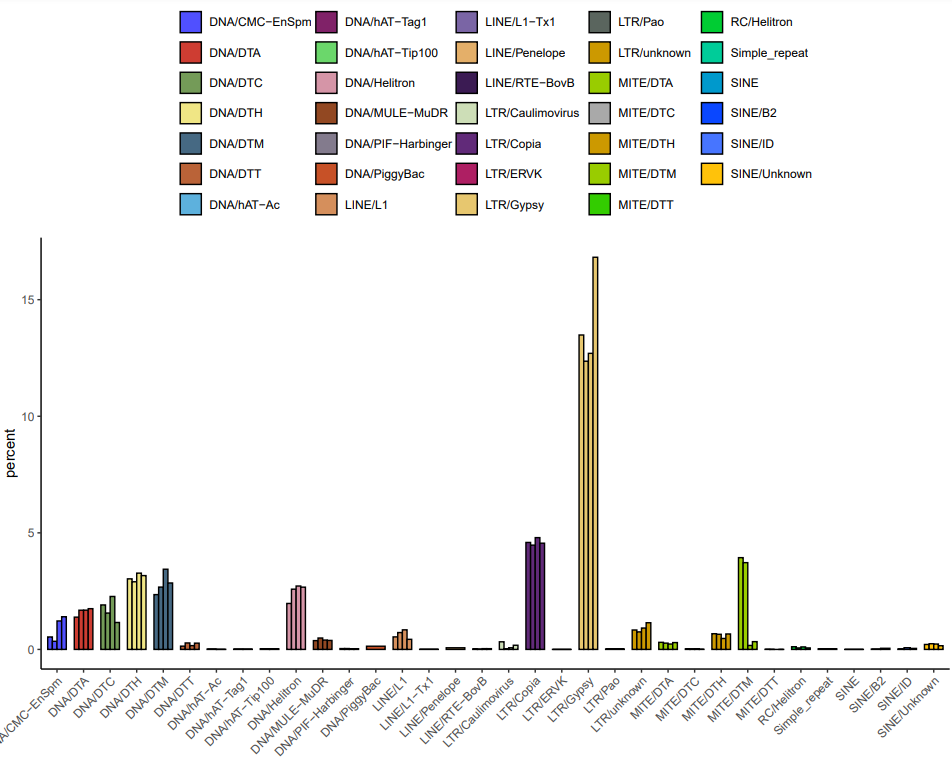


**Figure S6 The percent of different types of transposons in four *Prunus mume* (*P. mume* ‘LE_hap1’, *P. mume* ‘LE_hap2’, wild *P. mume* and *P. mume* Tortuosa) genomes.** The horizontal axis represents different types of transposons, and the vertical axis represents the proportion of transposons in the genome. Different colors represent different types of transposons.


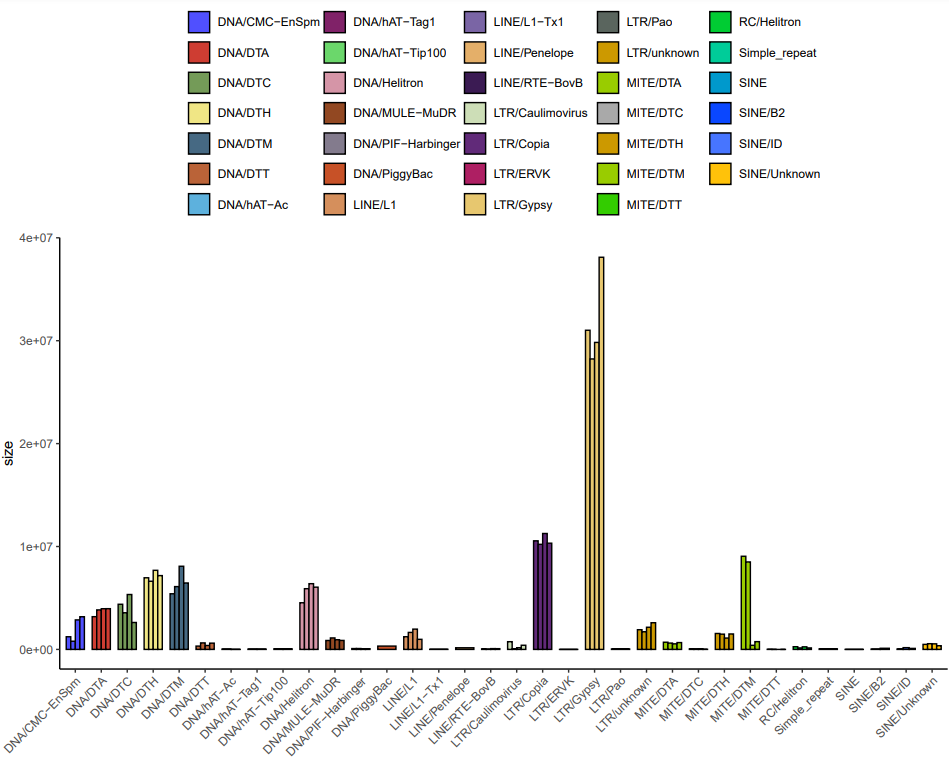


**Figure S7 The size of different types of transposons in four *Prunus mume* (*P. mume* ‘LE_hap1’, *P. mume* ‘LE_hap2’, wild *P. mume* and *P. mume* Tortuosa) genomes.** The horizontal axis represents different types of transposons, and the vertical axis represents the total length of transposons. Different colors represent different types of transposons.


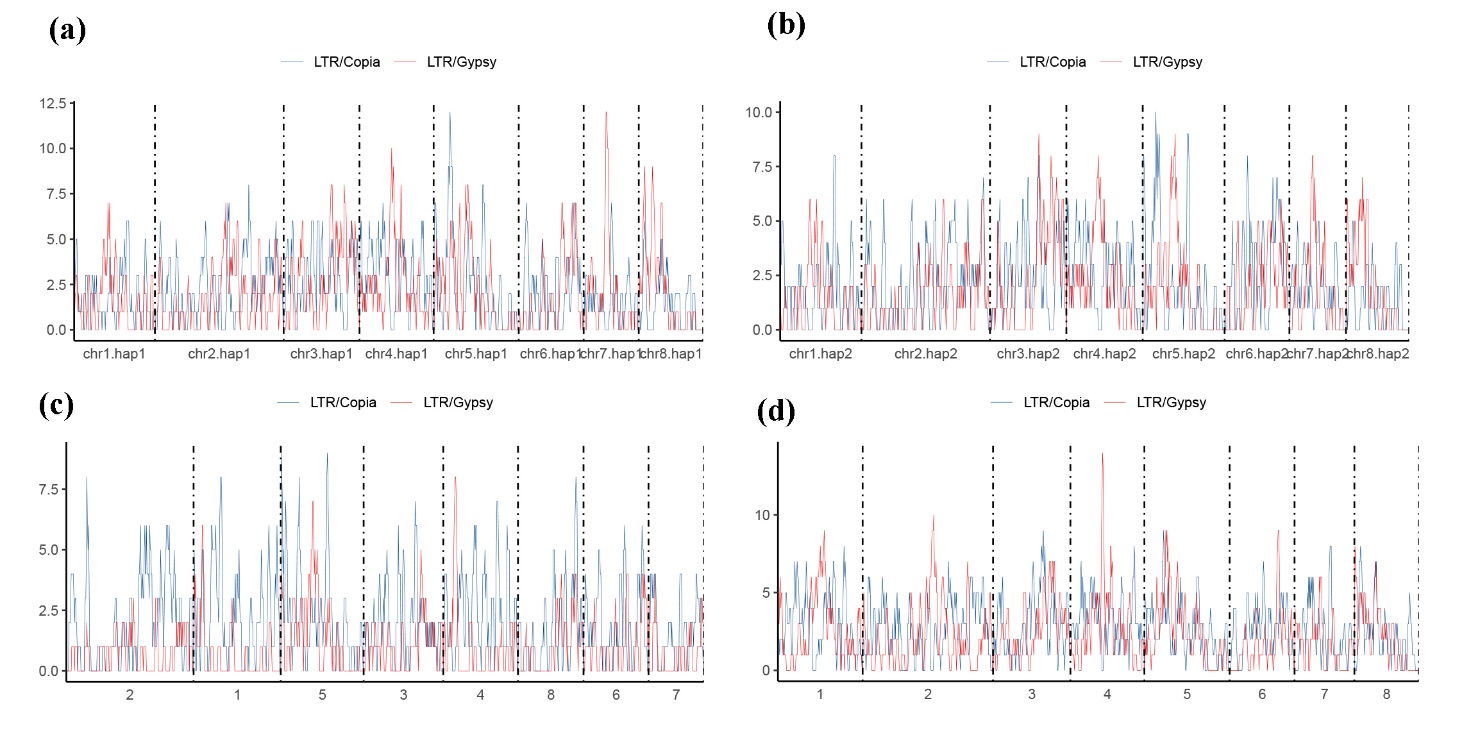


**Figure S8 Distribution of Gypsy and Copia type LTRs along chromosomes in the four *Prunus mume* genomes.** (a) *Prunus mume* LE_hap1**;** (b) *Prunus mume* LE_hap2; (c) *Prunus mume* Tortuosa; (d) wild *Prunus mume*. The horizontal axis represents the 8 chromosomes of the *Prunus mume* genome, and the vertical axis represents the number of LTR transposons.


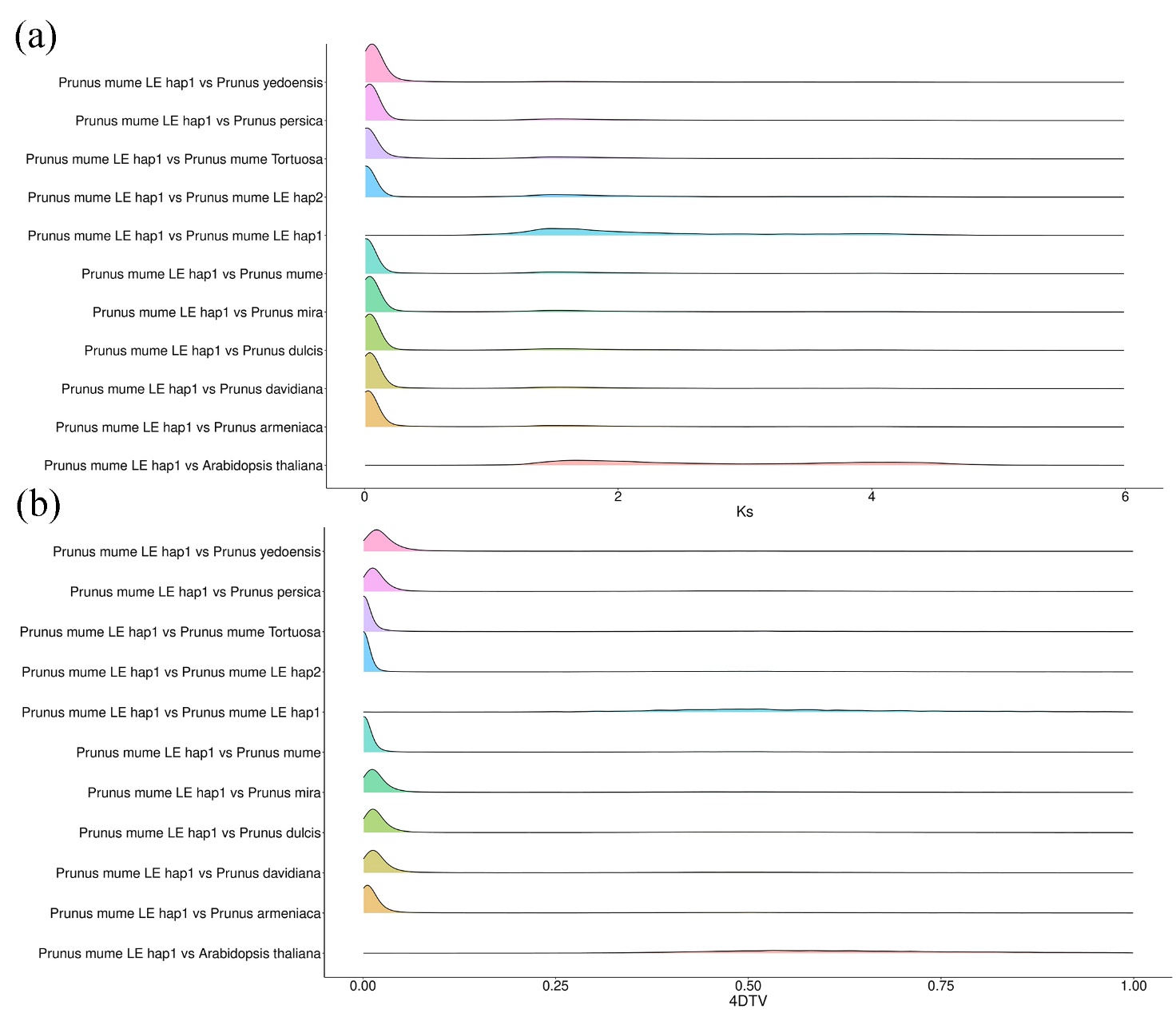


**Figure S9 The analysis of whole genome duplication.** (a) Horizontal coordinate indicates Ks, vertical coordinate indicates share (%); (b) Horizontal coordinate indicates 4DTV value, vertical coordinate indicates share (%). A total of 10 genomes and *Prunus mume* LE_hap1 were analyzed, including *Arabidopsis thaliana*, *Prunus armeniaca*, *Prunus davidiana*, *Prunus dulcis*, *Prunus kanzakura*, *Prunus mira*, *Prunus salicina*, *Prunus yedoensis*, *Prunus persica*, *Prunus mume Tortuosa*, *Prunus mume* Le_hap2.


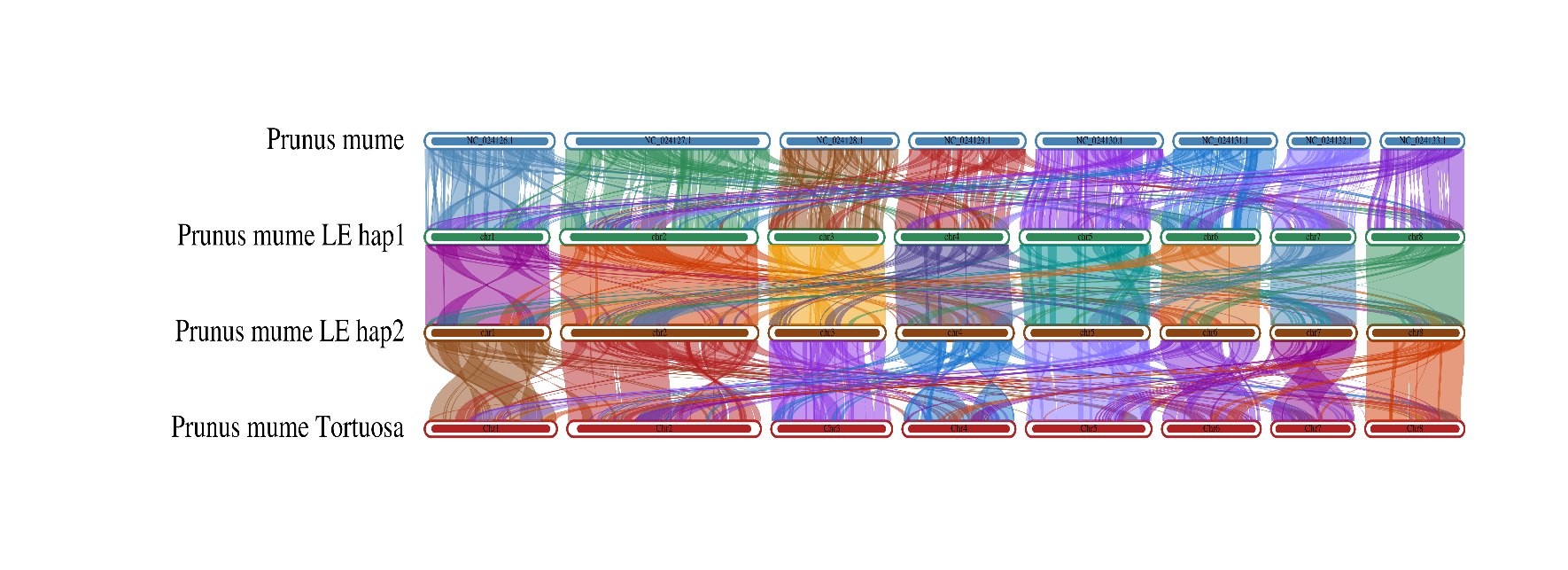


**Figure S10 Chromosome-level collinearity patterns among *Prunus mume* species.** The numbers indicate the pseudochromosome order generated from the original genome sequence. Each rounded rectangle represents a chromosome. Each color line represents 1 block. One block means that more than five paired genes were aligned in sequence.


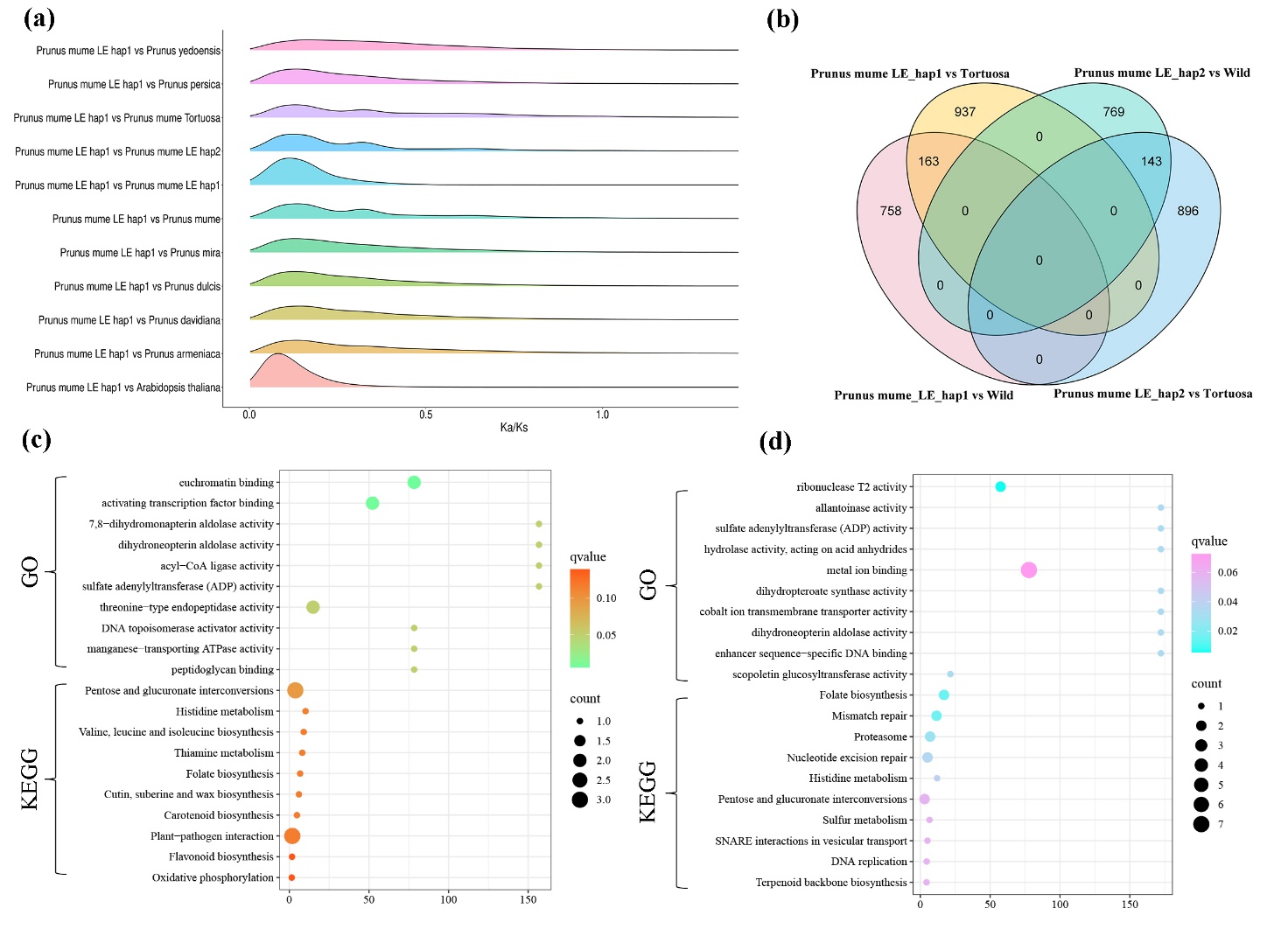


**Figure S11 Positive selection of genes and enrichment analysis.** (a)Analysis of non-synonymous mutation rate (Ka) and synonymous mutation rate (Ks) values. Horizontal coordinates indicate Ka/Ks, vertical coordinates indicate percentage (%), and lines of different colors indicate comparisons between species. (b) Venn plots of genes with Ka/Ks>1 in the *P. mume* LE_hap1 with wild *P. mume* and *P. mume* Tortuosa genomes and genes with Ka/Ks>1 in the *P. mume* LE_hap2 with wild *P. mume* and *P. mume* Tortuosa genomes. (c) GO and KEGG functional enrichment of the 163 shared genes in the *P. mume* LE_hap1 with wild *P. mume* and *P. mume* Tortuosa genomes. (d) GO and KEGG functional enrichment of the 143 shared genes in the *P. mume* LE_hap2 with wild *P. mume* and *P. mume* Tortuosa genomes. The size of the circle represents different numbers of genes, and the color of the circle represents different qvalues.


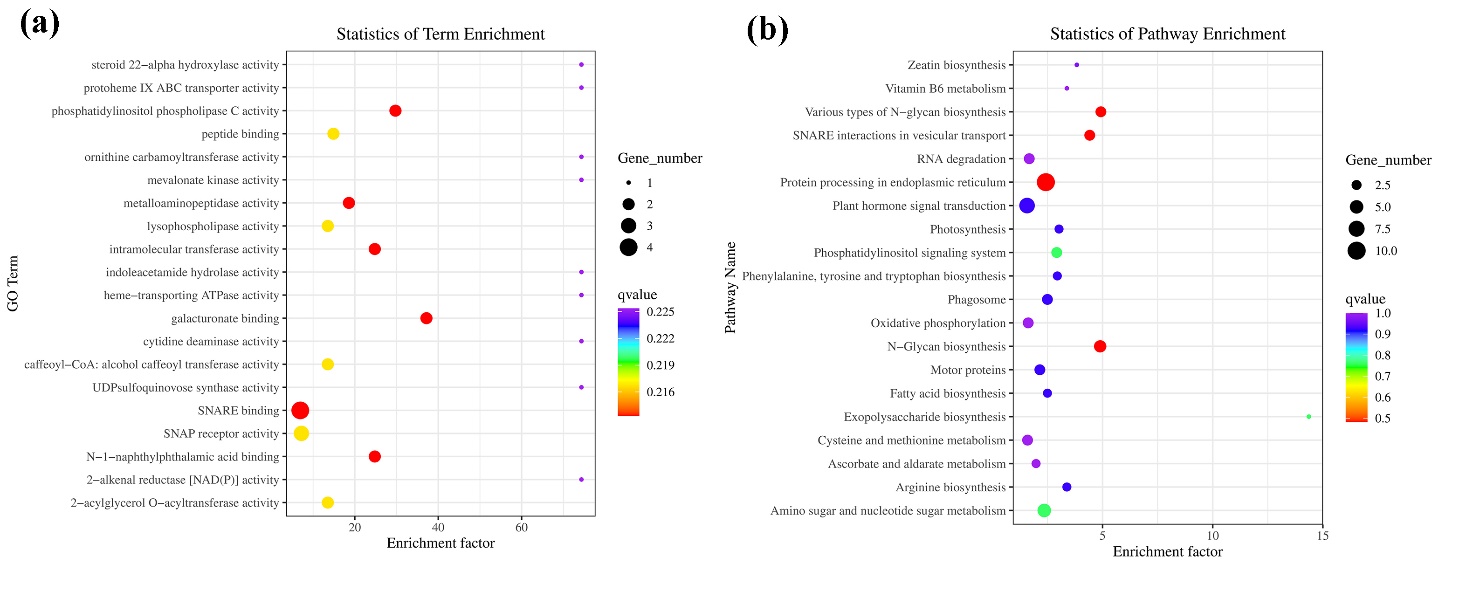


**Figure S12 The GO (a) and KEGG (b) pathway analysis of specific genes in the *Prunus mume* LE_hap1 genome.** The size of the circle represents different numbers of genes, and the color of the circle represents different qvalues.


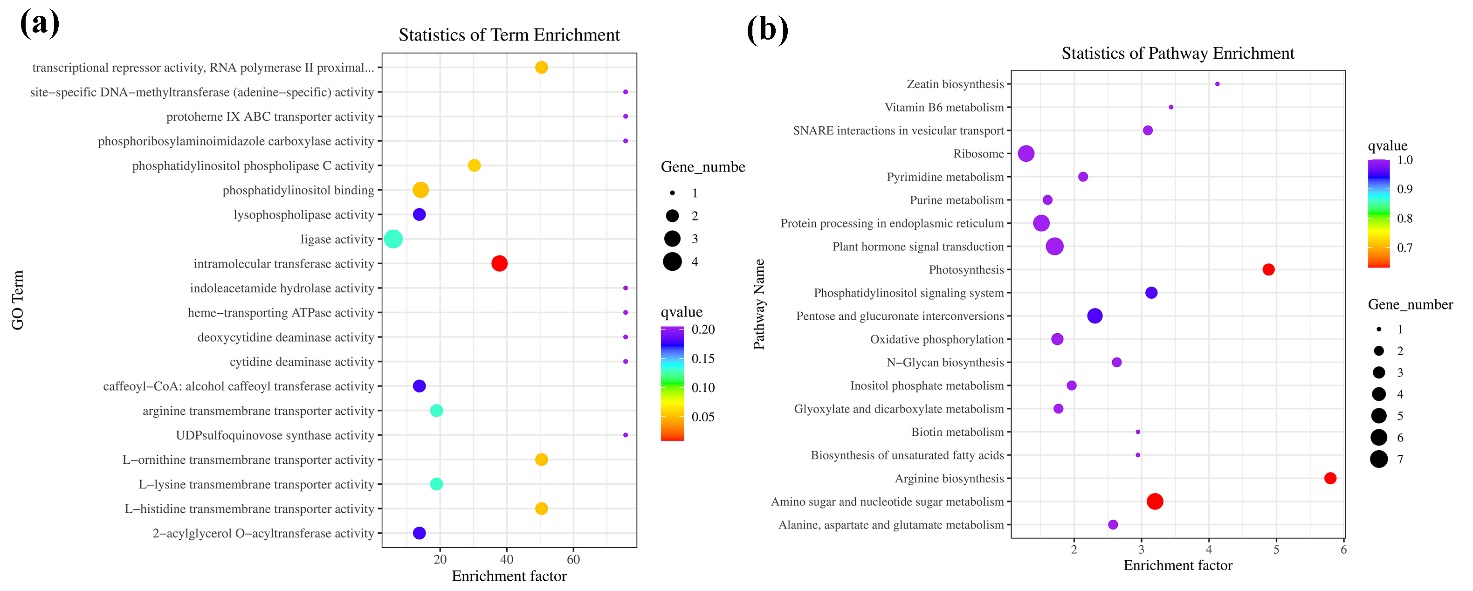


**Figure S13 The GO (a) and KEGG (b) pathway analysis of** **specific genes in the *Prunus mume* LE_hap2 genome.** The size of the circle represents different numbers of genes, and the color of the circle represents different qvalues.


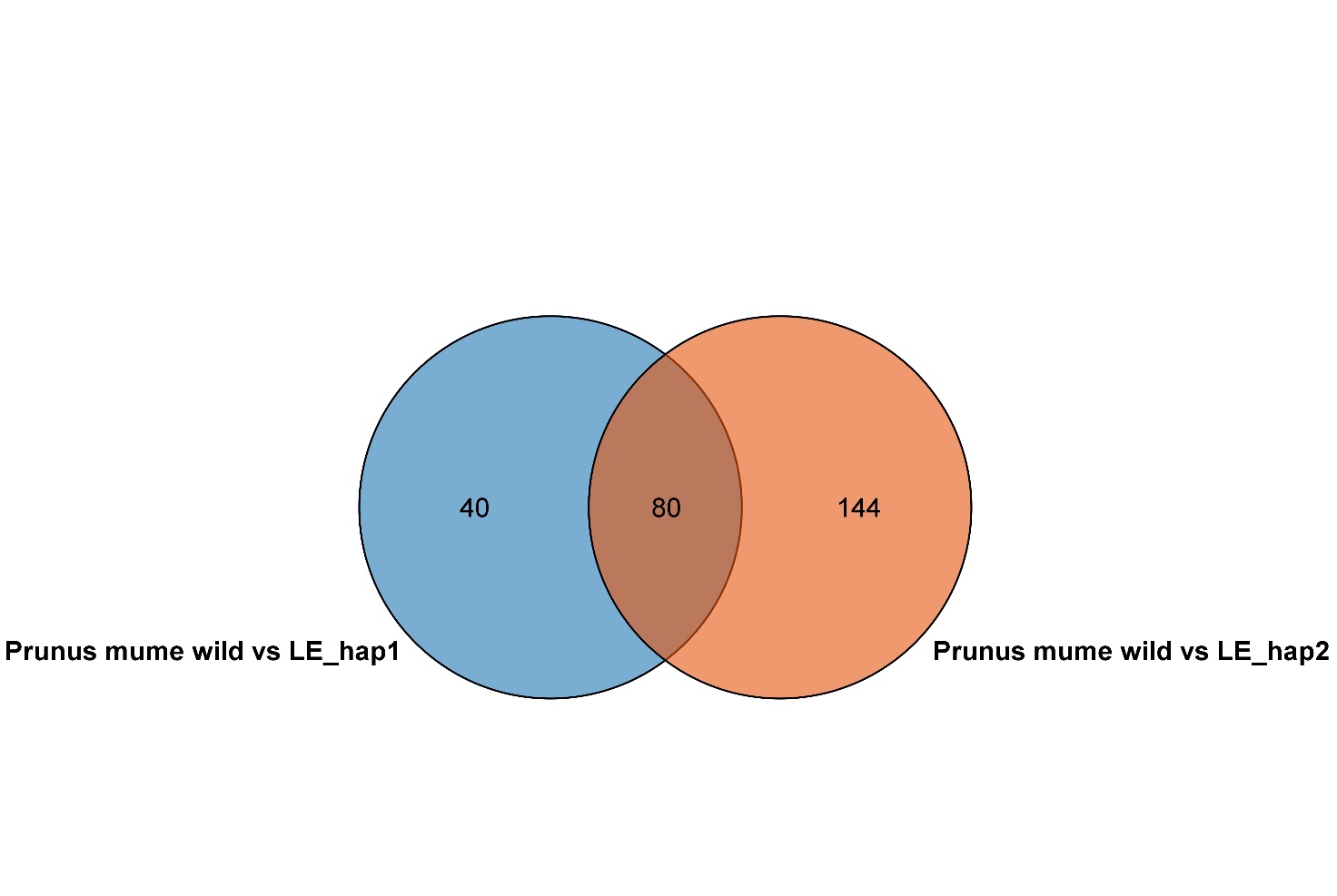


**Figure S14** **The specific genes in the wild *Prunus mume* genome, compared to the Prunus mume LE_hap1 and LE_hap2 genomes**


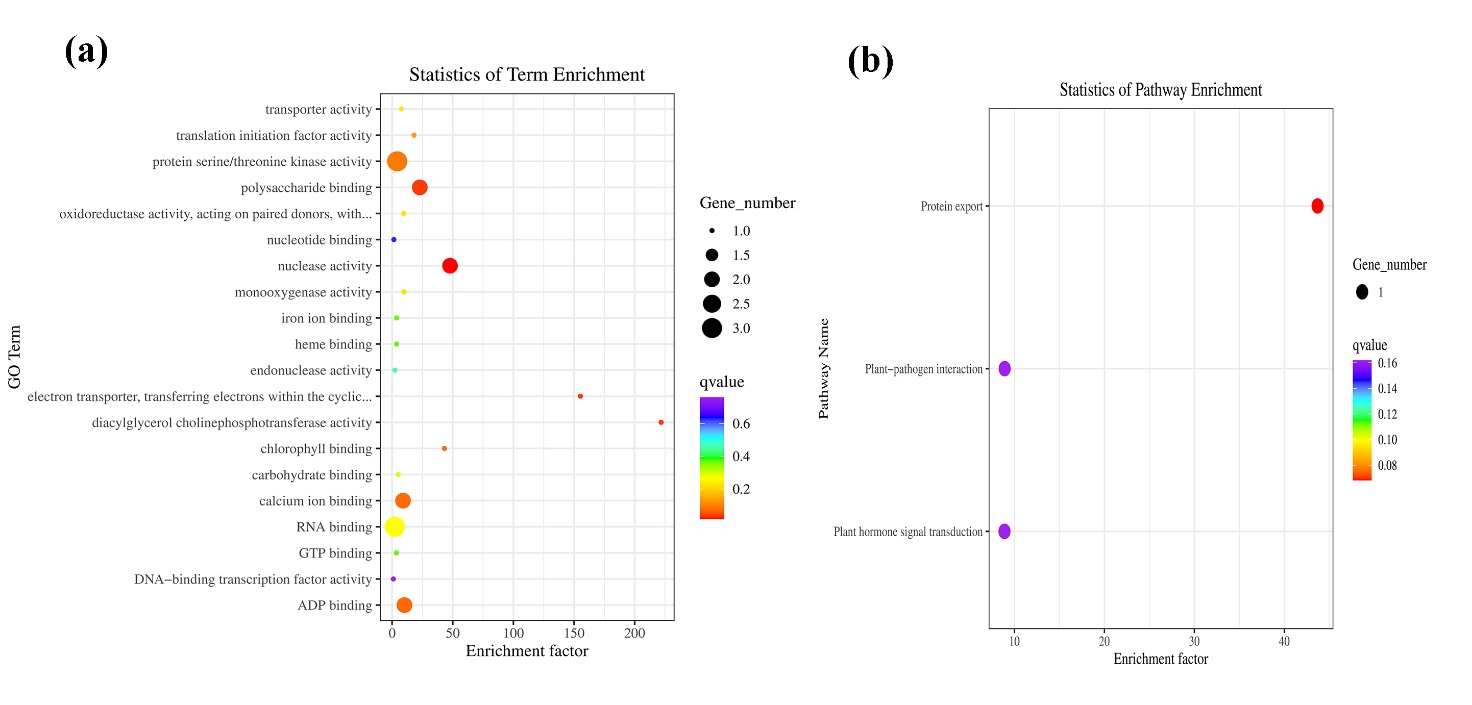


**Figure S15** **The GO (a) and KEGG (b) pathway analysis of specific genes in the wild *Prunus mume* genome.** The size of the circle represents different numbers of genes, and the color of the circle represents different qvalues.


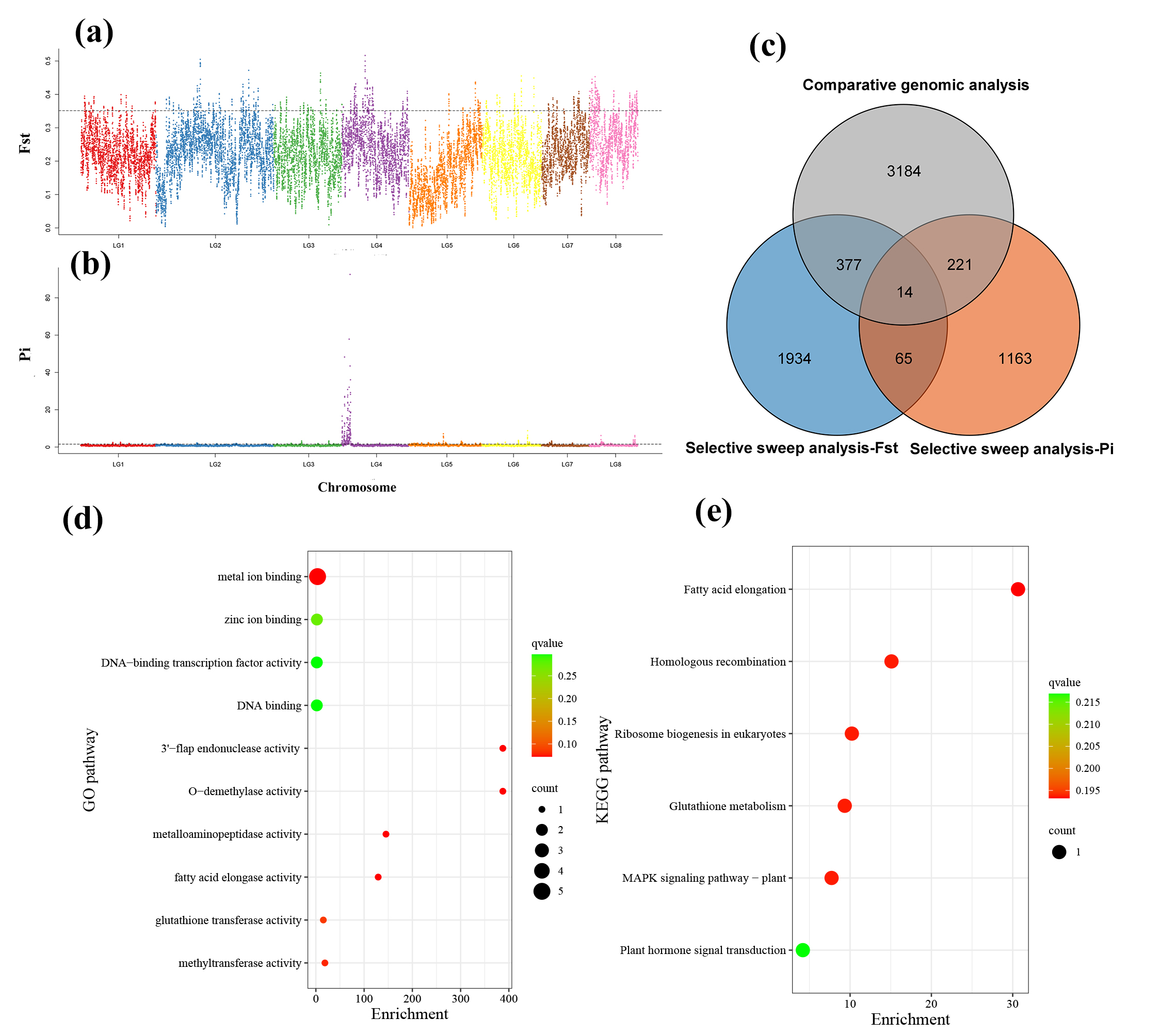


**Figure S16 Analysis of regions and genes under selection of populations at different phenotypes.** The distribution of F_ST_ (a) and Pi (b) values for the selective sweep analysis in different phenotypes groups, the position of the dashed line represents the top 5% of the selected areas. (c) Venn diagram showing the number of genes under selection in the two groups and comparative genomic analysis. Over-represented Gene Ontology (d) terms and Kyoto Encyclopedia of Genes and Genomes (e) pathways in overall selection. The size of the circle represents different numbers of genes, and the color of the circle represents different qvalues.


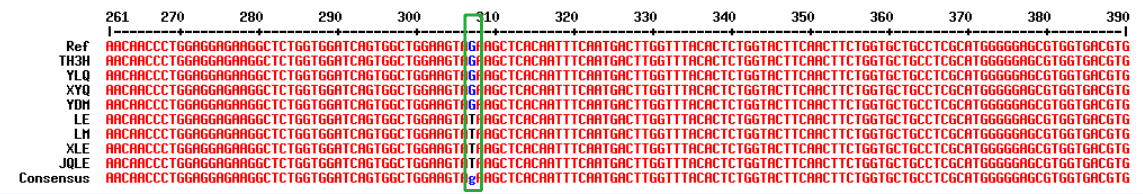


**Figure S17 The nucleotide sequence alignment of *PmGSTF2* gene in samples with low and high anthocyanin content groups.** The sequence TH3H, YLQ, XYQ and YDM represent the samples in the non-green sepal type group, sequence LE, LM, XLE and JQLE represent the samples in the green sepal type group, and the green box represents the mutation position. LE: Lv E; LM: Lv mei; XLE: Xiao Lve; JQLE: Jinqian Lve; TH3H: Taihu No.3; YDM: Yadan Mei; XYQ: Xiye Qing; YLQ: Yeli Qing.


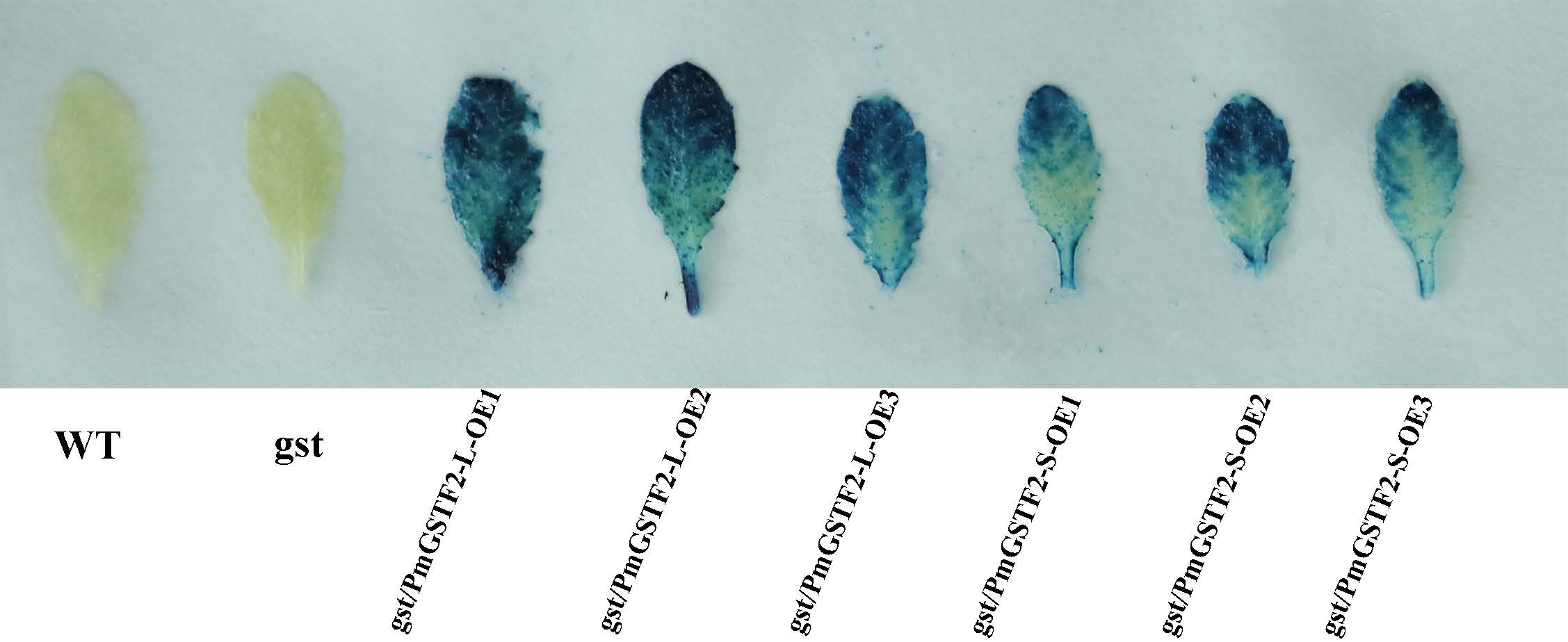


**Figure S18 The GUS staining of wild-type, mutant, and transgenic strains of *Arabidopsis thaliana***


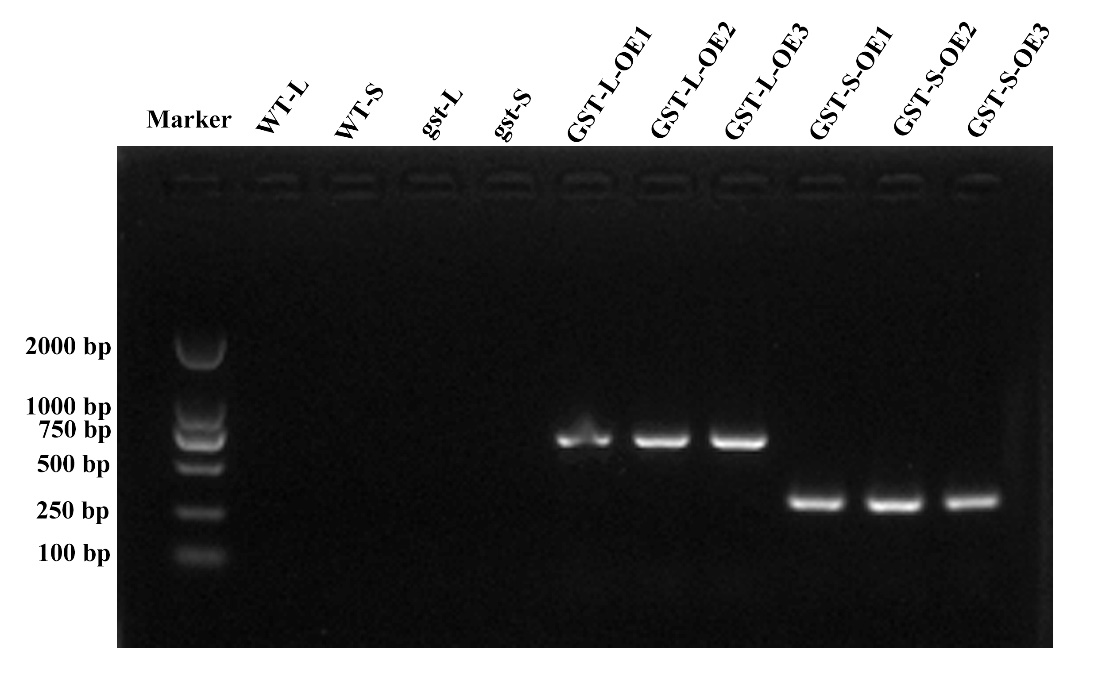


**Figure S19 The PCR amplification of transgenic, mutant, and wild-Type *Arabidopsis* cDNAs validates missing and complete *PmGSTF2* genes**


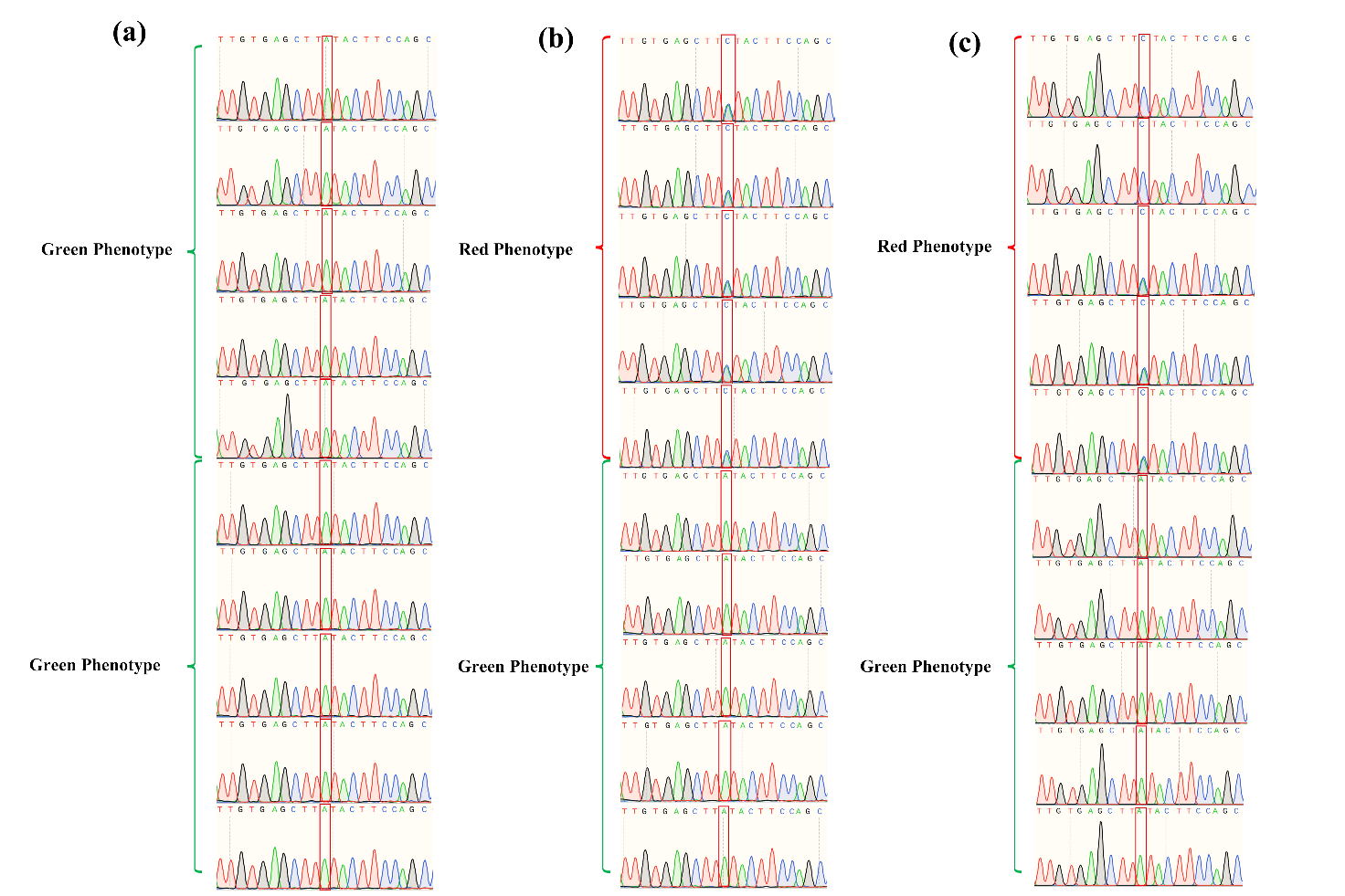


**Figure S20 The peak plot of sequencing results of *PmGSTF2* gene in F1 seedlings of different hybrid combinations.** (a) F1 seedlings in the hybrid combination of *P. mume* f. viridicalyx and *P. mume* Xiao Lve, the nonsense mutation sites of *PmGSTF2* gene in all green phenotype samples exhibit a single peak of A base**; (b)** F1 seedlings in the hybrid combination of *P. mume* f. viridicalyx and *P. mume* Yanglao No.2, the nonsense mutation sites of *PmGSTF2* gene in all green phenotype samples exhibit a single peak of A base, and in all red samples exhibit A/C base heterozygosity peak**; (c)** F1 seedlings in the hybrid combination of *P. mume* Yanglao No.2 and *P. mume* Gucheng No.1, the nonsense mutation sites of *PmGSTF2* gene in all green phenotype samples exhibit a single peak of A base; Some of the nonsense mutation sites in the *PmGSTF2* gene in the red phenotype samples exhibit a single peak at the C base, while others exhibit a heterozygous peak at the A/C base**.** The red box represents the site of nonsense mutation in *PmGSTF2* gene**.**


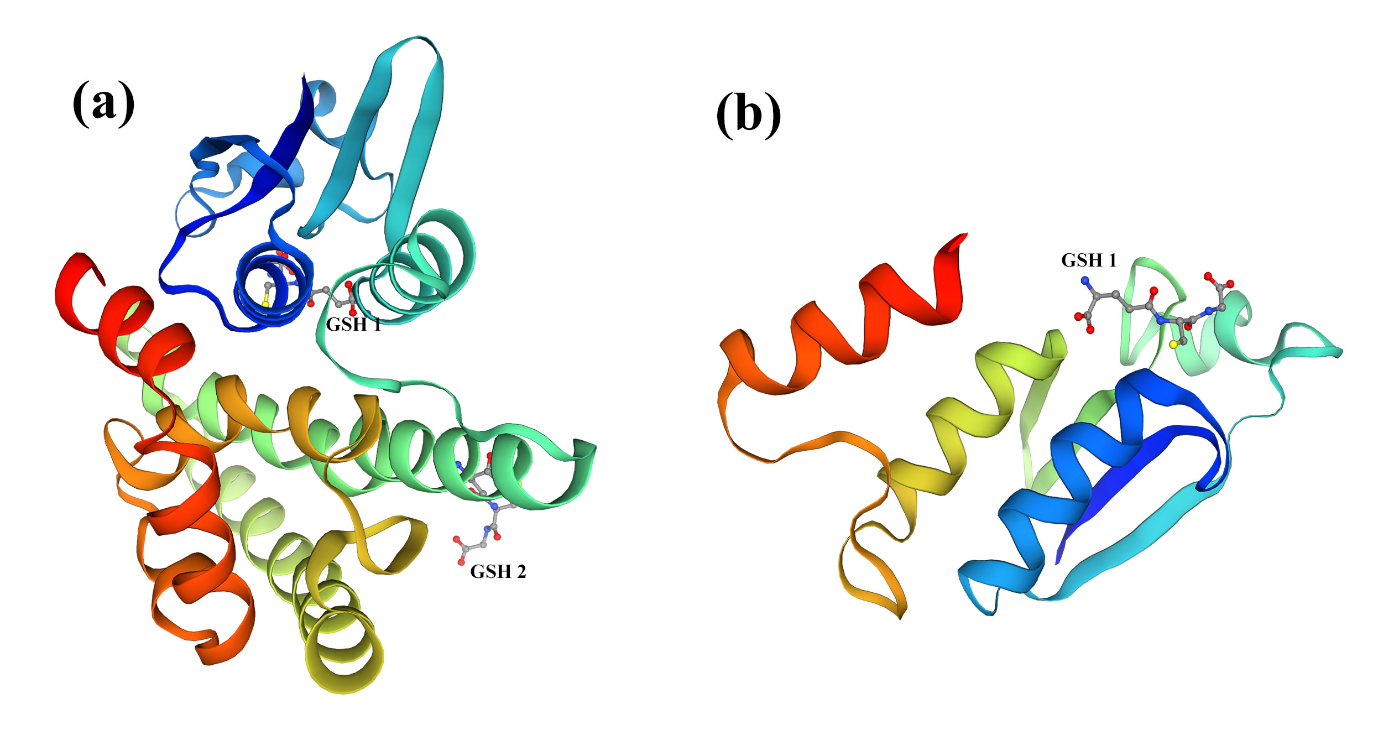


**Figure S21 The three-dimensional structure of *PmGSTF2* gene protein.** (a) The structure of *PmGSTF2*-L; (b) The structure of *PmGSTF2*-S.
